# How Sophisticated Are Neural Networks Needed to Predict Long-Term Nonadiabatic Dynamics?

**DOI:** 10.1021/acs.jctc.4c01223

**Published:** 2024-11-14

**Authors:** Hao Zeng, Yitian Kou, Xiang Sun

**Affiliations:** † Shanghai Frontiers Science Center of Artificial Intelligence and Deep Learning, NYU Shanghai, 567 West Yangsi Road, Shanghai 200124, China; ‡ State Key Laboratory of Precision Spectroscopy, East China Normal University, Shanghai 200062, China; ¶ Division of Arts and Sciences, NYU Shanghai, 567 West Yangsi Road, Shanghai 200124, China; § NYU-ECNU Center for Computational Chemistry at NYU Shanghai, 3663 Zhongshan Road North, Shanghai 200062, China; ∥ School of Computer Science and Technology, East China Normal University, Shanghai 200062, China; ⊥ Department of Chemistry, New York University, New York, New York 10003, United States

## Abstract

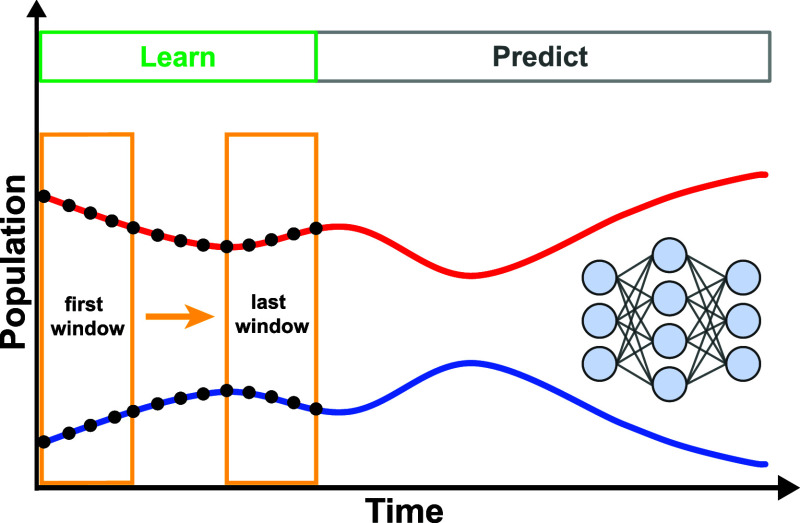

Nonadiabatic dynamics is key for understanding solar energy conversion and photochemical processes in condensed phases. This often involves the non-Markovian dynamics of the reduced density matrix in open quantum systems, where knowledge of the system’s prior states is necessary to predict its future behavior. In this study, we explore time-series machine learning methods for predicting long-time nonadiabatic dynamics based on short-time input data, comparing these methods with the physics-based transfer tensor method (TTM). To understand the impact of memory time on these approaches, we demonstrate that non-Markovian dynamics can be represented as a linear map within the Nakajima-Zwanzig generalized quantum master equation framework. We further propose a practical method to estimate the effective memory time, within a given tolerance, for reduced density matrix propagation. Our predictive models are applied to various physical systems, including spin-boson models, multistate harmonic (MSH) models with Ohmic spectral densities and for a realistic organic photovoltaic system composed of a carotenoid-porphyrin-fullerene triad dissolved in tetrahydrofuran. Results indicate that the simple linear-mapping fully connected neural network (FCN) outperforms the more complicated nonlinear-mapping networks including the gated recurrent unit (GRU) and the convolutional neural network/long short-term memory (CNN-LSTM) in systems with short memory times, such as spin-boson and MSH models. Conversely, the nonlinear CNN-LSTM and GRU models yield higher accuracy in the triad MSH systems characterized by long memory times. These findings offer valuable insights into the role of effective memory time in non-Markovian quantum dynamics, providing practical guidance for the application of time-series machine learning models to complex chemical systems.

## 1 Introduction

Quantum dynamical processes that occur among multiple electronic states are known as nonadiabatic dynamics, which are fundamentally responsible for charge and energy transfer in photochemistry, photosynthesis, solar energy conversion, and quantum information.^1−6^ In a typical theoretical description of nonadiabatic dynamics in open quantum systems, the electronic degrees of freedom (DOF) are treated as the system, and the nuclear DOF as the bath. It should be noted that the electronic DOF in the electronic-nuclear (system-bath) partitioning is not a standalone physical entity compared with a solute molecule embedded in a solvent environment, thus open quantum system should be distinguished from the thermodynamic open system that has a clear spacial boundary between the system and the surroundings. For this electronic-nuclear composite system, a complete description is given by the overall density matrix defined in the basis corresponding to the direct product of electronic and nuclear Hilbert spaces. The evolution of the overall density matrix is governed by the quantum Liouville equation, which describes closed quantum systems. The quantum Liouville equation implies that the quantum dynamics of the composite system is Markovian since a full knowledge of the current status will determine the future status.

If one is interested in the electronic dynamics of the same composite system given in the system-bath form, the electronic information is characterized by the reduced density matrix (RDM) obtained by tracing out the nuclear DOF of the overall density matrix. The evolution of RDM can be formulated in terms of the Nakajima-Zwanzig generalized quantum master equation (GQME) with a projection superoperator that filters the RDM information and projection-free inputs that can be approximate or exact time correlation functions.^7−20^ In this case, the system dynamics will generally be affected by the bath, and the influence of the bath on the system is expressed with memory kernels. The memory kernels reflect the non-Markovian effects, which means that a history of the RDM dynamics rather than just the current status would be required to know the future status of the system. The generic descriptions using Markovian dynamics of full knowledge of closed quantum systems versus non-Markovian dynamics of partial knowledge of open quantum systems give a choice of perspective on the same composite systems. The non-Markovianity is the price that one has to pay when only partial knowledge of the composite system is known and to predict. In other words, the future of RDM dynamics cannot be predicted easily without knowing the history of RDM dynamics. For example, the transfer tensor method (TTM) has been used to construct non-Markovian transfer tensors in a black-box fashion and then predict long-time RDM dynamics using short-time dynamics with different initial conditions as input.^21−25^ Thus, both GQME and TTM approaches would require the knowledge of short-time nonadiabatic dynamics as input to generate the long-time nonadiabatic dynamics.

The nonadiabatic dynamics are challenging to simulate because of the intertwined electronic and nuclear DOF. It is true that “numerically exact” quantum-mechanical solutions can be obtained for some model systems using hierarchical equations of motion (HEOM),^26^ multilayer multiconfiguration time-dependent Hartree (ML-MCTDH),^27,28^ time-dependent density matrix renormalization group (TD-DMRG),^29−31^ and tensor-train methods.^32−35^ However, these “numerically exact” approaches may struggle to converge in certain parameter regimes and are often computationally expensive.^34^ A more practical kind of numerical methods is approximate trajectory-based mixed quantum-classical and semiclassical dynamics, such as mean-field Ehrenfest,^36^ fewest-switches surface hopping (FSSH),^37^ linearized semiclassical (LSC)^38−40^ and symmetrical quasiclassical (SQC)^41,42^ dynamics based on the mapping Hamiltonian. It can still be burdensome to directly apply these approximate nonadiabatic dynamics methods in complex condensed-phase systems on a large spatiotemporal scale.

Recently, machine-learning (ML) techniques have been successfully applied to many areas of data-driven applications, such as computer vision, natural language processing, autonomous driving, and so on. Especially, physics-informed neural networks have been shown to provide great utilities in solving partial differential equations.^43−45^ Besides the fruitful application of artificial neural networks (ANN) for potential energy surfaces of molecular and condensed-phase systems,^46−48^ time-series ML tools have been directed to represent the time evolution of quantum states.^49,50^ For Markovian dynamics, neural-network quantum states (NQS) were proposed to present the restricted Boltzmann machine (RBM) inspired variational wavefunction of many-body quantum systems,^51^ and their applications in solving Lindblad quantum master equation.^52,53^ Besides, Markovian dynamics of the full knowledge of nonadiabatic dynamics including the electronic mapping variables and the nuclear phase space variables within the symmetrical quasiclassical (SQC) dynamics based on the Meyer-Miller mapping Hamiltonian^54^ were shown to be captured by a long short-term memory (LSTM) model.^55^

In contrast, non-Markovian dynamics would be expected if we focus on the nonadiabatic dynamics of open quantum systems and only partial knowledge is given such as the RDM dynamics. In this case, there are several kinds of ML models that can be distinguished in terms of what information is known in training and how to use the trained model. When the ML models are trained with the dynamics of all time of interest, typically transfer learning to another physical system or dynamics starting from different initial conditions can be predicted. For example, fully connected neural network (FCN) model, a type of ANN with simple network structure, was trained to propagate the quantum dynamics of a 1-dimensional Heisenberg model starting from a new initial condition.^56^ Also, the convolutional neural networks (CNN) model was trained with multiple early and late RDM dynamical pieces of a particular length for spin-boson models starting from different initial conditions, and the ML model could predict the RDM dynamics starting from a new initial condition.^57^

The other way to leverage ML for non-Markovian dynamics is to predict long-time dynamics with short-time input, which is referred to as the short-for-long (SFL) ML models. The benefits of SFL ML models include reducing the computational cost for obtaining long-time dynamics directly and helping understand the non-Markovian effects in various systems. Here, the short time is compared with the interested time scale such as a few picoseconds for the photoinduced charge transfer process, and if the dynamical computation scales linearly with the simulated time, it is straightforward to estimate the reduced computational cost by ML. The SFL ML approaches can be further categorized into two types: one is trained with a single RDM trajectory and the other is with multiple RDM trajectories due to different electronic initial conditions. The recently emerged ML models using kernel ridge regression,^58,59^ LSTM,^60^ hybrid convolutional neural network/long short-term memory (CNN-LSTM)^61^ are all examples of the Type-1 SFL ML for nonadiabatic dynamics. Among these techniques, CNN-LSTM has the most sophisticated neural network structures and has been shown to give accurate predictions in model systems.^61^ A recent data-driven Markovian embedding reconstruction of non-Markovian dynamics could also determine the size of the effective environment and denoise the system dynamics.^62^ The Type-2 SFL ML models along with the TTM/GQME methods are trained with multiple trajectories starting from different electronic initial conditions. An important difference between TTM/GQME and the Type-2 SFL ML models is that the TTM and GQME require the RDM trajectories whose initial RDM can form a complete basis while ML models could work with arbitrary initial electronic conditions, such as an electronic population on a particular state. For example, FCN model was shown to capture the time-local generators of open quantum systems by training with multiple trajectories of the reduced quantum dynamics.^63^ Is seems that both of the simple FCN and the sophisticated CNN-LSTM have successful cases in predicting quantum dynamics, but is there a difference in their applicable regions?

Before we answer the above question, there is a significant problem we need to address first when applying these SFL ML methods for nonadiabatic dynamics of open quantum systems: the length of sufficient learning time cannot be known *a priori*, which should in principle cover the memory time of the non-Markovian dynamics. Of course, when the exact quantum-mechanical dynamics are available (that is only possible in simple model systems), the memory time can be determined by a convergence test with an increasing length of learning time. In most realistic scenarios, exact quantum dynamics are impossible to obtain, and even for approximate nonadiabatic dynamics, long-time dynamics can be expensive to obtain and less accurate than short-time dynamics. Therefore, we need guidance on how to estimate the effective memory time and access the applicability of different SFL ML models as well as TTM and GQME approaches.

In this work, we aim to develop a practical way to estimate the effective memory time for SFL ML models that are trained with a short-time single trajectory as well as the physics-based TTM/GQME approach and try to provide some guidelines on ML model selection. We will start with the formal expressions of Nakajima–Zwanzig GQME to show the reduced system propagation is a linear mapping with sufficient long learning time, and then propose a practical strategy to estimate the memory time for RDM propagation in non-Markovian dynamics. Several ML approaches including FCN, LSTM,^64^ CNN-LSTM,^65^ gated recurrent unit (GRU)^66^ will be applied to different physical model Hamiltonians, such as the spin-boson models (SBM),^67^ the multistate harmonic (MSH) models with an analytical spectral density,^68^ and the MSH models constructed for photoinduced charge transfer in realistic organic photovoltaic carotenoid-porphyrin-fullerene triad dissolved in tetrahydrofuran solvent.^69^ We will explore the linear-mapping and nonlinear-mapping ML models’ applicability in physical model systems that have various memory lengths, and test whether long-memory systems can be captured by nonlinear-mapping ML methods like the GRU and CNN-LSTM networks.

The remainder of this paper is organized as follows. Section 2 describes the linear mapping properties of nonadiabatic dynamics from GQME and propose the memory time estimation scheme. Section 3 presents the machine-learning approaches for predicting long-time dynamics with short-time input. Section 4 describes the three different physical model Hamiltonians for nonadiabatic dynamics in complex and condensed-phase systems. Section 5 reports and discusses the results. Section 6 provides the concluding remarks.

## 2 Theory

### 2.1 Linear Mapping from Generalized Quantum Master Equation

We start from the quantum Liouville equation (QLE) that governs the evolution of the overall density matrix ρ̂(*t*) in the composite system that is defined within the electronic (system) and the nuclear (bath) Hilbert spaces and characterized by Hamiltonian *Ĥ*:
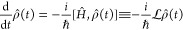
1The equation of motion for the reduced density matrix, σ̂ = Tr_N_(ρ̂) obtained by taking the trace over the nuclear Hilbert space, can be formulated by introducing the projection superoperators  and its complementary superoperator :
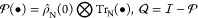
2where ρ̂_N_(0) is the initial nuclear density matrix and  is the identity superoperator. Applying the projection superoperators on both sides of QLE, we can obtain the Nakajima–Zwanzig (NZ) equation:^7−9,18^
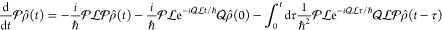
3The second term in NZ equation is the inhomogeneous term, which can be eliminated by assuming the initial density matrix to be a product state, i.e., ρ̂(0) = ρ̂_N_(0) ⊗ σ̂(0), and the NZ equation is simplified as^15^
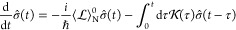
4where the time-local propagator of the system is  and the memory kernel that reflects the non-Markovian effects is defined as
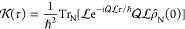
5Let us consider the discretized times that are separated by Δ*t* and denote objects at discretized steps using subscript such as σ̂(*n*Δ*t*) = σ̂_*n*_ and , then eq 4 can be integrated to be
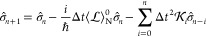
6Here, the time step size Δ*t* is expected to be small enough to avoid significant discretization error in RDM dynamics, which should be up to *O*(Δ*t*^2^), and the comparisons of different time step sizes for selected models are shown in Supporting Information.

We introduce non-Markovian propagator 
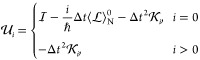
7such that the discretized RDM is given by
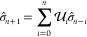
8Next, we show that the non-Markovian propagator is a linear map. We revisit the definition of a linear map. A map  is *a linear map* if for any two vectors **x**, **y** ∈  and any scalar *c* the following conditions are satisfied:  and . From this definition, we find that all the discrete non-Markovian propagators  are linear maps, since trace, projection superoperators, and Liouvillian are all linear maps, so they can be represented by matrices. The above analysis demonstrates that there exist linear maps that describe the non-Markovian propagation of the reduced system by mapping the historical RDMs to the future RDM. In the language of TTM, the non-Markovian propagators are called the transfer tensors.^24^ Furthermore, realistic systems are expected to have a finite memory time denoted as *t*_mem_ = *M*Δ*t*, which means that  when *i* ≥ *M*. Thus, we introduce an integer *M* time step to the propagation of RDM when *n* > *M*:
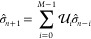
9This finite time step *M* or equivalently the memory time *t*_mem_ implies that the non-Markovian propagator can be represented by a matrix-vector multiplication, and can theoretically utilized as autoregression for infinite long RDM trajectory.

### 2.2 Memory Time Estimation for Reduced Density Matrix Propagation

We recast the propagation of RDM in eq 9 into the matrix-vector multiplication form **y** = **Ax**, where vector **y** ∈  is reshaped from RDM σ̂_*n*+1_ and vector **x** ∈  is reshaped from an array of RDMs (σ̂_*n*_, ···, σ̂_*n*–*M*+1_), thus matrix **A** ∈  is reshaped from .

Now, we estimate the error in the propagation if we truncate the input information after *K* recent RDMs that are less than the total *M*. To this end, we split the input vector **x** = **x**_0_ + **x**_1_, where the truncated input vector **x**_0_ = (σ̂_*n*_, ···, σ̂_*n*–*K*+1_, **0**_*n*–*K*_, ···, **0**_*n*–*M*–1_), which is set to zero after the *K*th RDM entry, and the complementary vector **x**_1_ = (**0**_*n*_, ···, **0**_*n*–*K*+1_, σ̂_*n*–*K*_, ···, σ̂_*n*–*M*–1_). It is straightforward to show that **x**_0_ and **x**_1_ are orthogonal to each other since **x**_0_ · **x**_1_ = 0. Similarly, we can split the propagation matrix **A** = **A**_0_ + **A**_1_, where **A**_0_ has all zeros in the last (*M* – *K*)*F*^2^ columns and **A**_1_ has all zeros in the first *KF*^2^ columns. It follows that **A**_0_**x**_1_ = **A**_1_**x**_0_ = **0**. We can express the propagation of RDM as below:
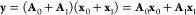
10Here, the first term **y**_0_ = **A**_0_**x**_0_ is the approximate RDM after the truncation, and the neglect of the second term **y**_1_ = **A**_1_**x**_1_ will cause the error. We define the truncation error as the ratio of the norms , and we expect the error to be less than a small tolerance ε, i.e., .

To estimate the effective cutoff length *K* with a given tolerance ϵ, we introduce two approximations.

1. Upper bound approximation, which assumes ∥**Ax**∥ ≈ *s* ∥**A**∥ · ∥**x**∥ and *s* is a scaling factor. From the definition of matrix norm induced by vector norm , we have ∥**Ax**∥ ≤ ∥**A**∥ · ∥**x**∥, and here we assume that the norm is approximately proportional to its upper bound.
2. Stationary norm approximation: ∥σ̂(*t*)∥ ≈ constant for any *t* ≥ 0. In this case, the reshaped RDM vectors (σ̂_*n*_, ···, σ̂_*n*–*M*–1_) have identical 2-norm, so .

Using these two approximations, we expect the relative error to be expressed as
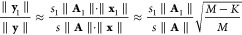
11When the above relative error is less than a tolerance ε, the following indicator *I*_ε_^*M*^(*K*) is expected to be less than 1:
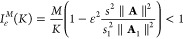
12The indicator provides a practical strategy to select the truncating length *K*: for a long enough length *M* and a given ε, the smallest *K* that satisfies *I*_ε_^*M*^(*K*) < 1 can be treated as an effective memory time and it is expected to give a result similar to *M* within error ε. The vector and matrix norms are derived above and shown in the result section as 2-norm defined as , which is the largest singular value of matrix **A**. We note that the Frobenius norm of the matrix  can be used to avoid calculating the spectral radius and give similar performance as shown in the Supporting Information.

## 3 Machine-Learning Approaches

### 3.1 Data Set Generation

In this work, the reference nonadiabatic dynamics is generated with resolution-of-identity linearized semiclassical dynamics type 2 (RI-LSC2)^40,68−70^ for different physical model systems detailed in Sec. 4 with a time step of 0.001 ps or reduced unit and 10^6^ trajectories were averaged in the simulation of the semiclassical dynamics in these physical model systems. It is noted that the ML strategies employed here are expected to work with many quantum dynamical methods as shown before,^61^ like the SQC, LSC, FSSH, Ehrenfest, spin-mapping, extended classical mapping models, as well as numerically exact HEOM, ML-MCTDH, TD-DMRG, tensor-train thermofield dynamics.^34^ The focus of ML approach is to learn and predict the given reference RDM dynamics, rather than generating the full quantum dynamics, so we will evaluate the performance of the ML models using the mean squared error (MSE) from the reference dynamics. Consider general *F*-state physical system, the RDM σ̂ is *F* × *F* Hermitian matrix, which include *F* real diagonal elements σ_*ii*_(*i* = 1, ···, *F*) or the populations, and *F*(*F* – 1)/2 complex off-diagonal elements or the coherences that is equivalent to *F*(*F* – 1)/2 independent numbers considering the Hermitian property σ_ij_^*^ = σ_*ji*_. Thus, there are *F*^2^ features or the independent variables of RDM at time *t* = *n*Δ*t* and Δ*t* = 0.01 ps or reduced unit, which include *F* populations and *F*(*F* – 1)/2 real and imaginary parts of the coherences denoted as vectorized  = {σ_*ij*_(*n*Δ*t*)|1 ≤ *i* ≤ *j* ≤ *F*} corresponding to the upper triangle matrix of the RDM.

The RDM dynamics from time 0 to the learning time *t*_*L*_ = (*L* – 1)Δ*t* is provided as the training set  that will be used to train and validate ML models. Suppose the effective memory time corresponds to *K* RDM sequence (*K* < *L*), we can slice the sequence of length *L* from the training set into *L* – *K* short sequences of length (*K* + 1). The *n*th short window of length (*K* + 1) is divided into the past information  and the current information . The set {(**X**_*n*_, **y**_*n*_)|*n* = 0, ···, *L* – *K* – 1} contains input and output pairs for training ML models, and the ML prediction also takes the same dimensions for input and output.

### 3.2 Fully Connected Neural Network

**Figure 1:**
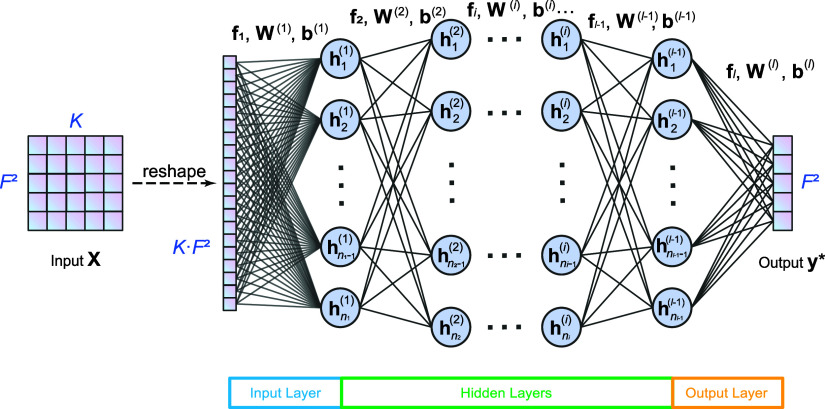
Illustration of fully connected neural network (FCN), a typical artificial neural network, for learning the reduced density matrix (RDM) dynamics of an *F*-state system using *K* previous steps, **X**, and predicting the RDM of the next time step, y*. In this work, the employed FCN uses a linear activation function and no hidden layer.

As a typical artificial neural network, the fully connected network consists of single or multiple fully connected layers and an *l*-layer FCN can be expressed by
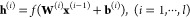
13where the input and output vectors are **x** ≡ **x**^(0)^ ∈ ^*n*^ and y* ≡ **h**^(*l*)^ ∈ ^*m*^, respectively; the weights and bias of the *i*th layer are **W** ∈ ^*m*×*n*^ and **b** ∈ ^*m*^, respectively; function *f*: ^*m*^ → ^*m*^ is the activation function, which may introduce nonlinearity to the neural networks. The network structure of FCN is shown in Figure 1, and the original input is a matrix **X**_*k*_ which is converted to a vector **x**_*k*_ by concatenating the RDM of all time steps. We have tested several commonly used activation functions, such as the linear function *f*(*x*) = *x*, the sigmoid function , the hyperbolic tangent function , and the Rectified Linear Unit (ReLU) function *f*(*x*) = max (0, *x*). We found that the linear function yielded the best results when FCN are built with the same data set, which aligns with the linear transformation property for the non-Markovian dynamical mapping that we show in the previous section.

### 3.3 Convolutional Neural Network/Long Short-Term Memory

**Figure 2:**
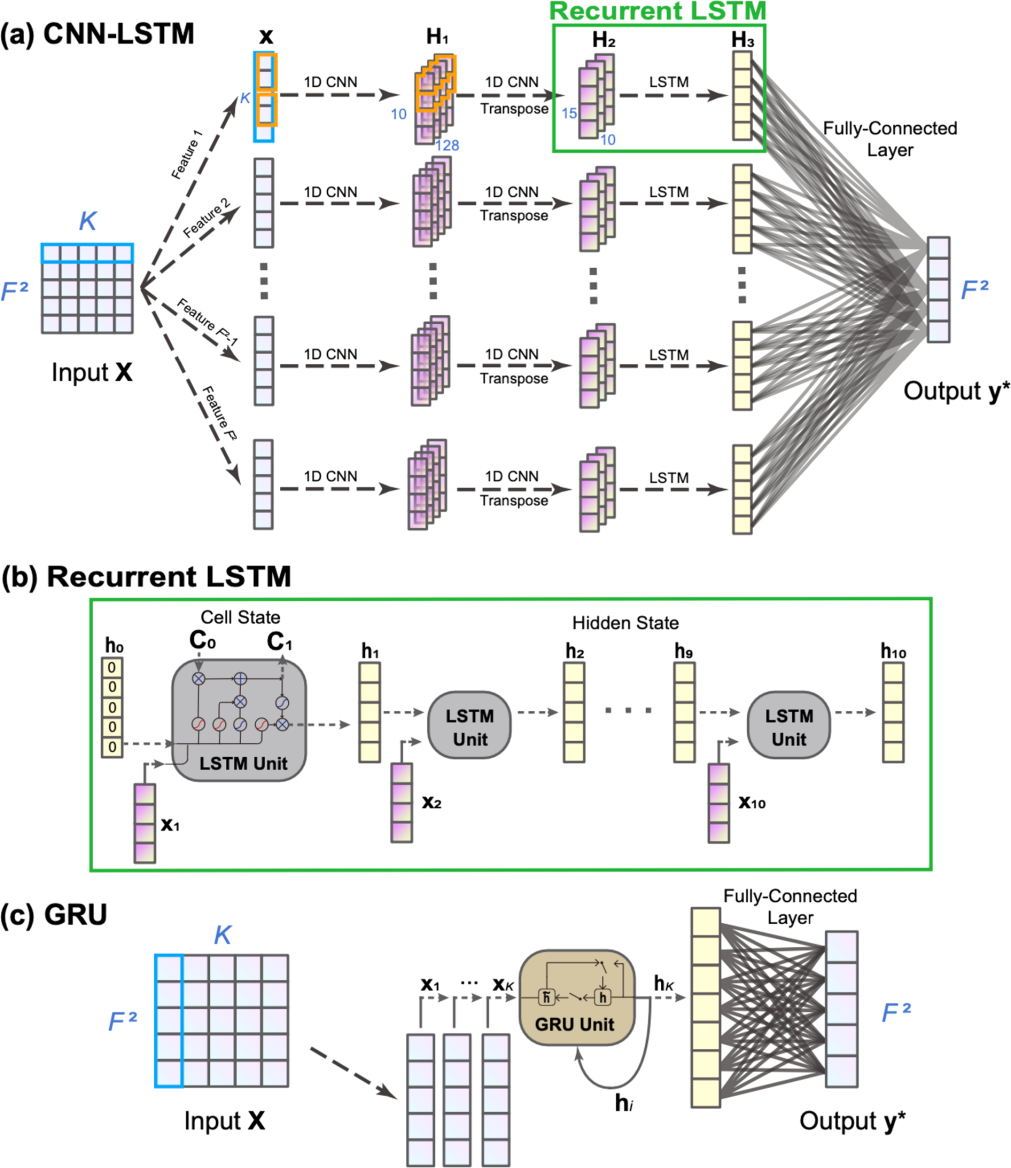
Illustration of the hybrid convolutional neural network/long short-term memory (CNN-LSTM) network (a), the inside structure of an LSTM unit (b), and the gated recurrent unit (GRU) network (c) for RDM of next time step using the RDM of *K* previous steps.

CNN and LSTM networks are two powerful types of neural networks that can be combined to leverage their respective strengths in time-series prediction. Here, we employ CNN-LSTM for feature-wise information extraction, which provides a nonlinear transformation, and use a fully connected layer for the final output. The network structure is shown in Figure 2a, where the input matrix **X** ∈ ^*F*^2^×*K*^ is separated into *F*^2^ feature sequences of time length *K*, and each feature sequence will be processed with a CNN-LSTM unit. The current CNN-LSTM networks are slightly different from our previous implementation, where the real and imaginary parts of coherence are combined and then used as the input for a CNN-LSTM unit,^61^ while the current model treats the real and imaginary parts of coherence separately. As shown in Figure 2a, each feature sequence, for example, the *j*th feature **x**_*j*_ of length *K*, undergoes the first convolutional layer with 128 one-dimensional kernels of length (*K*/10) and stride (*K*/10), resulting in a matrix **H**_1_ ∈ ^10×128^. Next, each row of **H**_1_ is passed through the second convolutional layer that consists of 15 kernels with length 128 and stride 1, giving rise to a matrix **H**_2_′ ∈ ^10×15^ and thus its transpose **H**_2_ ∈ ^15×10^. Then, the 10 columns of **H**_2_ are fed to the recurrent LSTM layer (see Figure 2b), whose hidden states **h**_0_, ···, **h**_10_ have *n*_L_ hidden units (here we use *n*_L_ = 128), resulting in the final hidden state **H**_3_ = **h**_10_ ∈ ^*n*_L_^ after 10 recurrent cycles. Finally, the outputs of *F*^2^ LSTM units are combined into a single vector of dimension *n*_L_ × *F*^2^, which connects with the outcome that predicts the RDM of the next time step y* ∈ ^*F*^2^^ through a fully connected layer.

### 3.4 Gated Recurrent Unit

The gated recurrent unit (GRU) is a type of recurrent neural network (RNN) for time-series data and also requires fewer parameters than LSTM, making it computationally efficient while still being effective in capturing long-term dynamics. A GRU network with *n*_G_ hidden units propagates the state vector **x**_*t*–1_ at time *t* – 1 to **x**_*t*_ at the next time step *t* in the following way. The hidden state of the previous time step **h**_*t*–1_ is also required as input. The propagation of one time step using GRU includes four steps: get update gate **z**_*t*_, reset gate **r**_*t*_, candidate hidden state **h̃**_*t*_, and final hidden state **h**_*t*_, which are expressed as
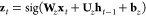
14
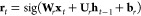
15
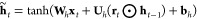
16
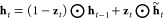
17Here, **W**_*z*_, **W**_*r*_ ∈ ^*n*_G_×*F*^2^^ and **U**_*z*_, **U**_*r*_ ∈ ^*n*_G_×*n*_G_^ are the weight matrices (here we use *n*_G_ = 128), **b**_*z*_, **b**_*r*_ ∈ ^*n*_G_^ are the bias terms, and sig(*x*) is the sigmoid activation function. The element-wise product is denoted as ⊙. The update gate **z**_*t*_ ∈  and the reset gate **r**_*t*_ ∈  describe how much of the previous hidden state should be remembered and forgotten, respectively. the candidate hidden state **h̃**_*t*_ ∈  represents the new information influenced by the reset gate. The initialization of the hidden state **h**_0_ ∈  is set to be zero. After *K* recurrence of GRU, the final hidden state **h**_*K*_ ∈  is obtained, which will be directed to a fully connected layer to produce the final output RDM of next time step y* ∈ 

### 3.5 Loss Function with Regularized Population

Throughout this work, we used the mean square error with regularized population (MSE-RP) as the loss function for the ML models. The physical constraint is the total population should be one, or ∑_*j*=1_^*F*^ σ_*jj*_ = 1. The MSE-RP loss function for predicted RDM σ^(*p*)^ and reference RDM σ^(*r*)^ with a regularization hyperparameter α is given by
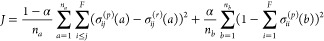
18where *n*_*a*_, *n*_*b*_ are the numbers of predicted data points, although they are not necessarily a time sequence. For ML training purposes, *n*_*a*_ = *n*_*b*_ and they correspond to the same training or validation set. The first term is the MSE term that measures the error of the predicted RDM from the reference and the second term is the RP term that reinforces the conservation of the total population to be one.

### 3.6 Learning Time and Hyperparameter Optimization

In this work, we treat all the known dynamics for ML as the learning time *t*_learn_, which is the early time dynamics for SFL type of ML models, and the predicted long-time dynamics as the predicted time *t*_pred_. Within the learning time, for example, we divide the first 80% as the training-validation set, and the last 20% as the hyper-learning time. The training and validation sets contain randomly sampled dynamical windows of length *K* and typically the training set weights 90% and the validation set weights 10% in the entire training-validation set. The training and validation sets are used in the optimization of neural network parameters. The optimization algorithm for these parameters is chosen to be Adam optimizer^71^ with an initial learning rate of 10^–5^. The hyper-learning time data set is for hyperparameter optimization, which improves the stability of the ML methods and reduces the human impact on the hyperparameter choice. The optimization algorithm for hyperparameters is a tree-structured Parzen estimator (TPE), which is is sequential model-based optimization approach.^72^ The hyperparameter optimization also utilizes the MSE-RP loss function (eq 18) except that the MSE term is based on the hyper-learning time data (*n*_*a*_) and the RP term is based on both the hyper-learning time and the predicted populations at long time (*n*_*b*_). This choice of loss function guarantees short-term dynamical accuracy and reinforces the long-time normalization condition of the total population. For the fixed learning time ML models, we optimize the three hyperparameters: (1) window size *K* for the non-Markovian dynamics, (2) loss tolerance for both ML training and validation, (3) regularization coefficient α in MSE-RP loss function. For the best performance ML models, we add the learning time *t*_learn_ to the hyperparameters that will be optimized. Since the hyperparameter surface is rather rugged, a single optimization may lead to a local minimum for the loss function, thus in practice, we select the best prediction from multiple trials of hyperparameter optimization.

## 4 Physical Model Hamiltonians

### 4.1 Spin-Boson Model

The spin-boson model has been widely used for understanding charge and energy transfer processes in molecular and condensed-phase systems.^67,73^ The spin-boson model Hamiltonian represents a system with two electronic states that are coupled to a collection of nuclear vibrational modes:
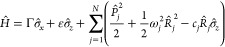
19where σ̂_*x*_ = |*D*⟩⟨*A*| + |*A*⟩⟨*D*| and σ̂_*z*_ = |*D*⟩⟨*D*|−|*A*⟩⟨*A*| are the Pauli matrices that are defined in the basis of the donor state |*D*⟩ and the acceptor state |*A*⟩; Γ is the electronic coupling coefficient; Δ*E* = −2ε is the reaction free energy; {*R̂*_*j*_, *P̂*_*j*_, ω_*j*_} = {*R̂*_*j*_, *P̂*_*j*_, ω_*j*_|*j* = 1, ···, *N*} are the mass-weighted coordinates, momenta and frequencies associated with the *N* nuclear normal modes, respectively; {*c*_*j*_} are the electronic-vibrational coupling coefficients. Here, {ω_*j*_, *c*_*j*_} are specified by discretizing the spectral density defined as
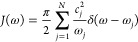
20In this work, we employ the Ohmic spectral density as below
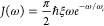
21where ξ is the Kondo parameter and ω_c_ is the cutoff frequency. The discretization scheme and the spin-boson model parameters of SBM #1–4 are described in the Supporting Information. The displacement between the donor and acceptor equilibrium geometry along the *j*th mode is *R*_*j*_^eq^ = 2*c*_*j*_/ω_*j*_^2^ (*j* = 1, ···, *N*). The reorganization energy between the donor and acceptor states is given by
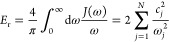
22

### 4.2 Multi-State Harmonic (MSH) Model

The MSH model Hamiltonian serves as a general extension of the two-level spin-boson model to multiple electronic states, and it can be constructed from all-atom MD simulations and quantum chemistry calculations introduced in ref (68). The effectiveness of the MSH model Hamiltonian has been demonstrated by the excellent agreement with the nonadiabatic dynamics obtained with the all-atom Hamiltonian.^69^ The general *F*-state MSH model Hamiltonian is given in the following form defined with interstate electronic diabatic couplings *V̂*_*XY*_ = Γ_*XY*_, (*X* ≠ *Y*):
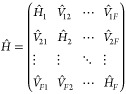
23where the nuclear Hamiltonians of the *F* electronic states are given by
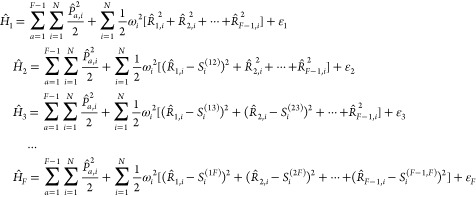
24Here, {ε_*X*_|*X* = 1, ···, *F*} are the potential energy minima of the *F* electronic states; {ω_*i*_|*i* = 1, ···, *N*} denote the *N* physical normal-mode frequencies of the entire system; {*R̂*_*a*,*j*_, *P̂*_*a*,*j*_|*j* = 1, ···, *N*; *a* = 1, ···, *F* – 1} are the mass-weighted coordinates and momenta associated with the *N* nuclear normal modes defined in the *F* – 1 extended dimensions such that the total nuclear DOF is *N*_*n*_ = (*F* – 1) *N*; {*S*_*i*_^(*aX*)^|1 ≤ *a* < *X* ≤ *F*} are the equilibrium shift components for the *X*th state’s PES along the *i*th mode in the *a*th subspace.

To construct the equilibrium shift components {*S*_*i*_^(*aX*)^}, the required molecular inputs are the energy-gap time correlation functions (TCFs) between all pairs of states obtained with all-atom simulations: *C*_*UU*_^(*XY*)^(*t*) = ⟨*U*_*XY*_(*t*) *U*_*XY*_(0)⟩ – ⟨*U*_*XY*_⟩^2^, where the energy gap between *X* and *Y* PESs is defined as *U*_*XY*_ = *V*_*X*_ – *V*_*Y*_. In the *F*-state MSH model, there are *F*(*F* – 1)/2 energy-gap TCFs {*C*_*UU*_^(*XY*)^(*t*)|1 ≤ *X* < *Y* ≤ *F*}. The corresponding reorganization energies between all pairs of states are given by
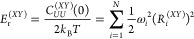
25where the equilibrium geometry distances between the PES minima of *X*, *Y* states, *R*_*i*_^(*XY*)^, are given by
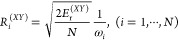
26The key property of the MSH Hamiltonian is that all the reorganization energy relationships are satisfied simultaneously such that the electronic-vibrational couplings in the multistate case are consistent, which has been demonstrated by the fact that the MSH model could reproduce the nonadiabatic dynamics obtained with all-atom Hamiltonian.^69^ The discretization of the spectral density from the energy-gap TCFs to obtain the frequencies is described in ref (68) and also the Supporting Information. Alternatively, if the spectral density is in a certain analytical form such as the Ohmic form in eq 21, the corresponding MSH model can also be constructed.

In this work, we consider five different MSH models that were parametrized for different conformations of the organic photovoltaic carotenoid-porphyrin-fullerene triad dissolved in tetrahydrofuran solvent, which are referred to as triad conf. 1–5.^68,74^ In addition, we consider two different MSH models with Ohmic spectral densities and anisotropic reorganization energies, referred to as MSH #1 and #2. The MSH model parameters are included in the Supporting Information.

## 5 Results and Discussion

In this section, we shall show the results of three ML approaches including FCN, GRU, and CNN-LSTM as well as the physics-based TTM approach for predicting long-time nonadiabatic dynamics with short-time data, which is the learning time set, in four spin-boson models (SBM #1–4), MSH models for triad conf. 1–5, and two MSH models with Ohmic spectral densities (MSH #1 and #2). Our focus is to have an understanding of how effective memory time would influence the performance of these SFL methods.

**Figure 3:**
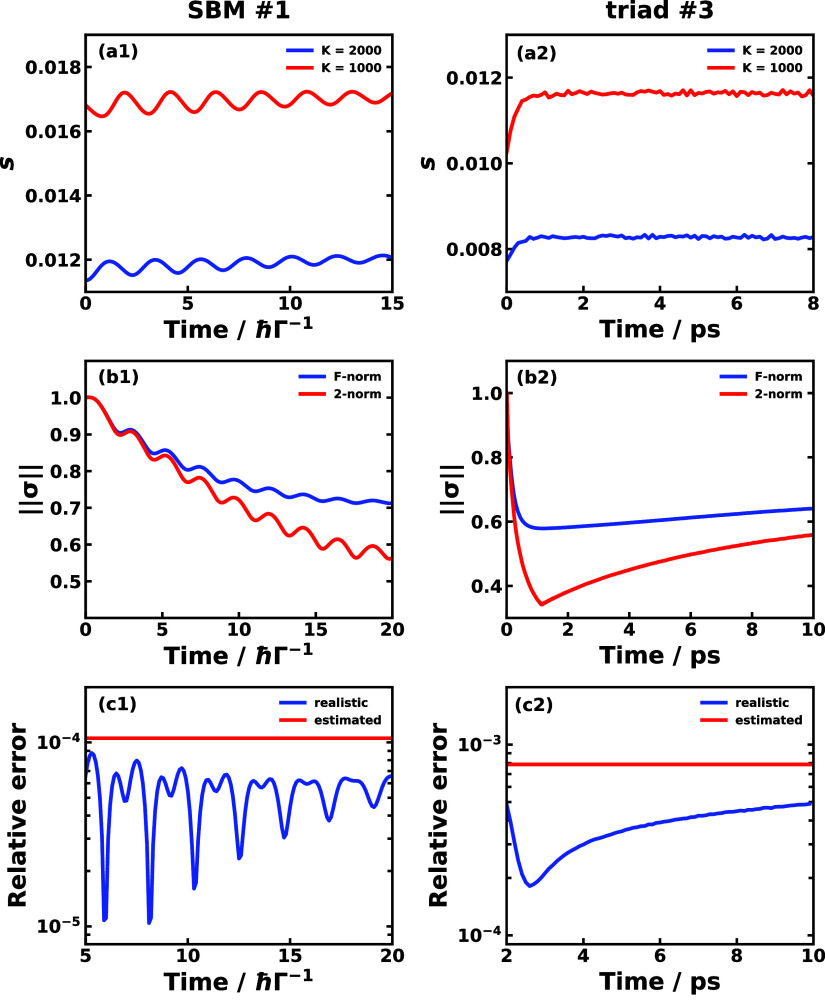
Support for approximations made in deriving effective memory time indicator as obtained from direct nonadiabatic dynamics simulations for spin-boson model #1 (left panels) and triad conf. Three (right panels). (a) coefficient ; (b) 2-norm and F-norm of the RDM; (c) comparison of relative truncation error from direct realistic simulations (blue) and estimated error using eq 11 (red).

First, we test the validity of the two approximations we employ in the derivation of the effective memory time estimation using the non-Markovian propagators given by TTM. Taking SBM #1 and triad conf. Three MSH model as examples, we show the support for these approximations in Figure 3. The upper bound approximation assumes that the coefficient  does not change much and can be justified by Figure 3a1,a2, where the *s* value in realistic nonadiabatic dynamics simulation stays rather stable (about 0.01–0.02) for a given truncating length *K* in both physical model systems. The stationary norm approximation of the RDM is supported by Figure 3b1,b2, where the F-norm of RDM ranges from 0.6 to 1.0 and the 2-norm ranges from 0.4 to 1.0. Moreover, the relative truncation errors obtained from direct realistic simulations are compared with the estimated error (eq 11) for both physical systems are shown in Figure 3c1,c2. It is evident that our estimated error poses an upper bound to the realistic truncation error in propagation, which endorses the effective memory time indicator *I*_ε_^*M*^(*K*).

The effective memory time indicator *I*_ε_^*M*^(*K*) depends on some knowledge of the long enough length *M* as shown in eq 12, and for a given *M*, the indicator less than 1 marks the effective truncating length *K* corresponding to the effective memory time. However, a practical issue is that one may not know the long enough length *M* for any given physical system to cover the entire memory time. So we need a strategy to systematically evaluate whether the *M* is long enough and then the truncating length *K* for a given tolerance ϵ could be meaningful.

**Figure 4:**
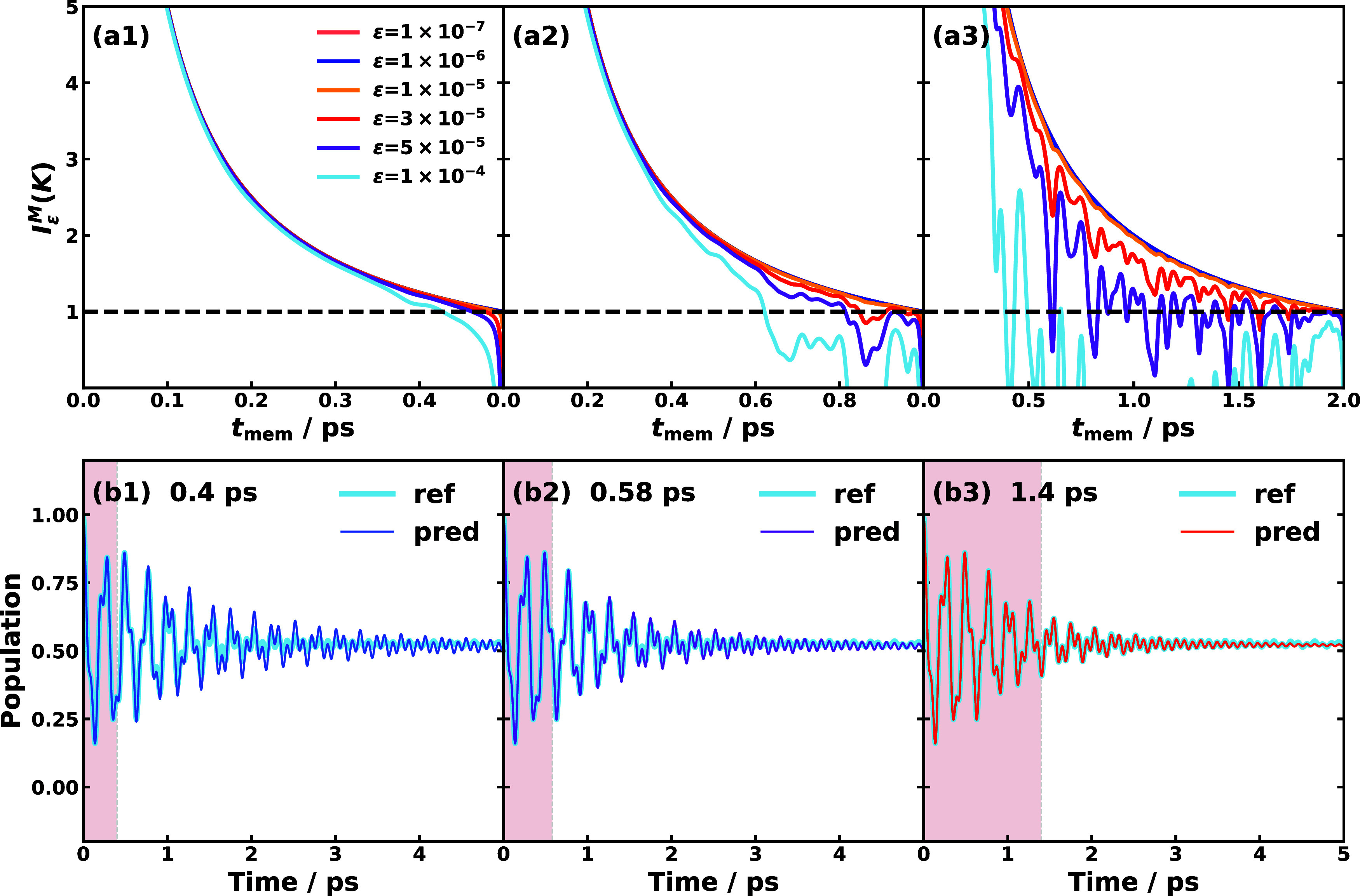
Effective memory time indicators *I*_ε_^*M*^(*K*) as a function of truncating length *K* for different max lengths *M* that correspond to 0.5 ps (a1), 1 ps (a2), and 2 ps (a3) for a variety of tolerance ϵ. The bottom panels are the TTM predictions of the |0⟩ population dynamics in MSH #1 model compared with reference RI-LSC2 dynamics with different truncating lengths *K* that correspond to learning time of 0.4 ps (b1), 0.58 ps (b2), and 1.4 ps (b3).

To this end, we propose to use the distribution of the indicator on different tolerance levels to determine if an *M* value is long enough. As shown in Figure 4a1–a3, we plot the indicator as a function of the truncating length *K* for MSH #1 on a variety of tolerance levels ranging from ε = 1 × 10^–7^ to ε = 1 × 10^–4^, and with different maximum length *M*. When *M* is short (0.5 ps in Figure 4a1), we observe that the smallest *K* value at which the indicator is less than 1 distributes heavily toward the end of *M* on a variety of tolerance levels. This means that 0.5 ps is not sufficient for obtaining the correct dynamics of this system, since the indicator and thus the propagator do not decay much until reaching the maximum length *M*. When *M* takes an intermediate value (1.0 ps in Figure 4a2), we start to see the wider distribution of the indicator on different tolerance levels such as 0.6 ps with ε = 1 × 10^–4^ and 0.8 ps at ε = 5 × 10^–5^, which means the effective memory time could be less than 1.0 ps. When *M* is long (2.0 ps in Figure 4a3), the distribution of the indicator is much broader than the other cases, and the shortest *K* values when the indicator is less than 1 range from 0.4 ps at ε = 1 × 10^–4^, 0.58 ps at ε = 5 × 10^–5^, and 1.4 ps at ε = 3 × 10^–5^. From our experience, the broad distribution of the indicator on different tolerance levels is the rule of thumb for a sufficient *M*. Thus, the effective memory time for MSH #1 model system should be within 0.6 ps at the recommended tolerance level of ε = 5 × 10^–5^. Of course, the tolerance should also depend on the time step size and the interested time scale for the dynamics. Figure 4b1–b3 shows the TTM predictions for MSH #1 model system with learning time 0.4, 0.58, and 1.4 ps, respectively. It is evident that the accuracy of the population dynamics prediction increases with the longer learning time.

**Figure 5:**
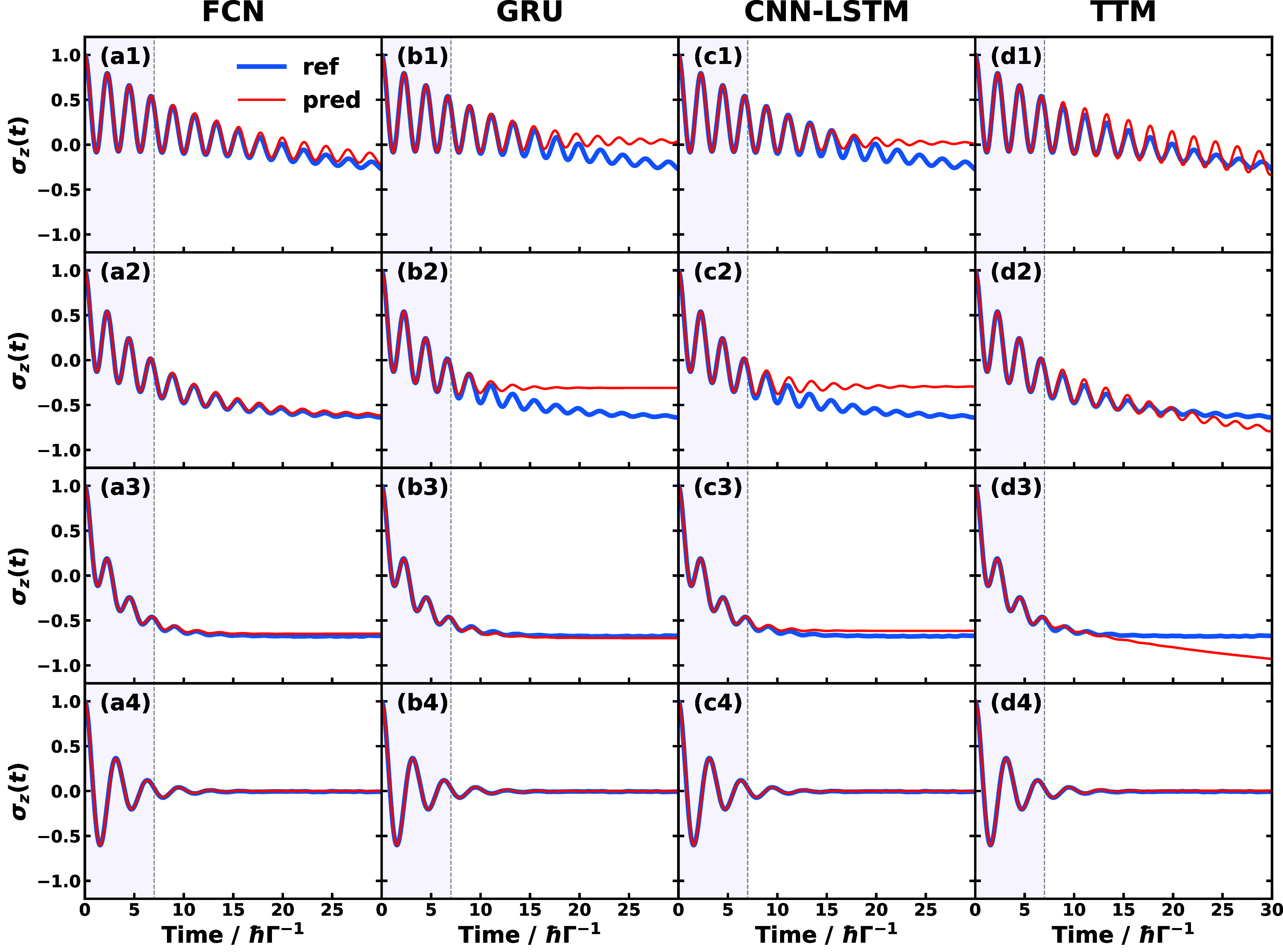
Fixed learning time SFL predictions for spin-boson models using different ML approaches including (a) FCN, (b) GRU, (c) CNN-LSTM, and (d) TTM approach. The four rows correspond to SBM #1–4, respectively. The predicted long-time dynamics of the population difference σ_*z*_(*t*) (red thin lines) are compared with the reference RI-LSC2 dynamics (blue thick lines). All the panels use a total learning time of 7 *ℏ*Γ^–1^, in which the last 1 *ℏ*Γ^–1^ is used as hyper-learning time. The effective memory time of SBM #1–4 is estimated to be 0.1, 0.1, 3, 3.5 *ℏ*Γ^–1^ with a tolerance of 10^–5^, respectively.

Next, we present a fair performance comparison of different ML SFL methods, all with the same fixed learning time, across spin-boson models, MSH constructed for triad conformations, and MSH with Ohmic spectral densities. The fixed-time comparison for the spin-boson models is shown in Figure 5, where all ML models including FCN, GRU, and CNN-LSTM are trained with the learning time 7 *ℏ* Γ^–1^, of which the last 1 *ℏ* Γ^–1^ is the hyper-learning time. From the parameters in Table S1, we can categorize SBMs as follows: (1) in terms of reaction free energy, SBMs #1–3 are asymmetric in the minimal potential energies on the donor and acceptor states and more specifically exothermic reaction (ε > 0), and SBM #4 is symmetric (ε = 0); (2) in terms of adiabatic and nonadiabatic regimes, SBM #1 is in the intermediate regime, while SBMs #2–4 fall into the nonadiabatic regime (ω_c_/Γ > 1); (3) in terms of Marcus regimes, SBM #1 is in the intermediate regime (−Δ*E* ≈ *E*_r_), SBM #2 and #3 are in the Marcus inverted regime (−Δ*E* > *E*_r_), and SBM #4 is in the Marcus normal regime (−Δ*E* < *E*_r_). The best-performed ML prediction is seen in the FCN method for all four SBMs, which features linear transformation. In contrast, GRU and CNN-LSTM featuring nonlinear transformation show a faster damping after 15 *ℏ* Γ^–1^ than the reference and underestimate the population decay in SBMs #1 and #2, but perform well with the other two SBMs. The physics-based TTM approach seems to show more inertia and less damped than the reference dynamics and it could reproduce the trend in SBMs #1, #2, and #4 but overestimates the population transfer in SBM #3. These results of SBMs are surprising at first glance since one would assume that if the learning time is long enough to cover the effective memory time, then we should expect both linear and nonlinear SFL approaches to work. The fact that the linear-mapping FCN captures better the nonadiabatic dynamics in SBMs than the other nonlinear-mapping ML strategies suggests that overfitting might be present in the more complicated nonlinear models if the intrinsic dynamics are linear. The linear-mapping TTM approach, on the other hand, directly propagates the system dynamics without fitting procedure, and thus is more sensitive to noise and inaccuracy in the input dynamics than FCN, making it prone to unstable predictions.

**Figure 6:**
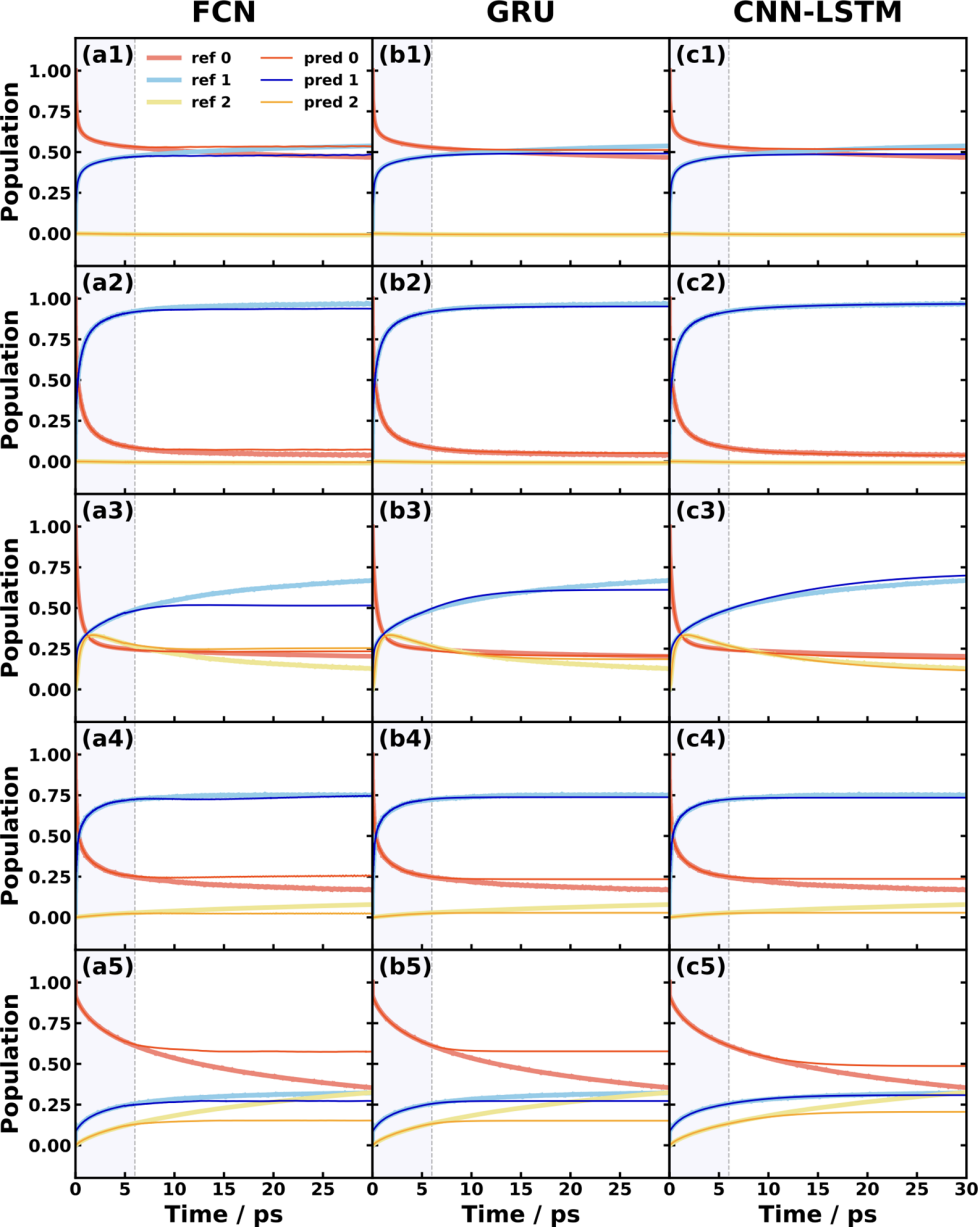
Fixed learning time SFL predictions for the triad MSH models using different ML approaches including (a) FCN, (b) GRU, and (c) CNN-LSTM. The five rows correspond to the triad conf. 1–5, respectively. The predicted long-time dynamics of the populations of three states (thin lines) are compared with the reference RI-LSC2 dynamics (thick lines). All the panels use a total learning time of 6 ps, in which the last 1 ps is used as hyper-learning time. The triad conf. 1–5 models are expected to have a memory time longer than 30 ps.

In Figure 6, we show the fixed-time performance comparison for the five triad conformations, which involve three electronic states: |0⟩ is the porphyrin-localized excited ππ* state, |1⟩ is the partially charge-separated state (CT1), and |2⟩ is the fully charge-separated state (CT2). The photoinduced population transfer dynamics initiated with the electronic population on the ππ* state while the nuclear distribution on the ground state shows an overdamped pattern because the triad dissolved in organic solvent contains a large amount of low-frequency modes and relatively large reorganization energy (0.2–1.5 eV). The prediction performance of all SFL methods is excellent in triad conf. 1 and 2 but show noticeable deviations of populations in triad conf. 3–5, where the linear-mapping FCN gives a faster damp than nonlinear-mapping GRU and CNN-LSTM. Among the three SFL methods, CNN-LSTM generates the best prediction for all triad conformations, although the fast-damping issue is observed in triad conf. 4 and 5. It is believed that the memory time of triad systems is so long that the linear propagation is not fully represented within the learning time 6 ps used here, but nonlinear-mapping models with more sophisticated structures could capture the dynamical effects. Thus, when the system has a rather long memory time, the nonlinear-mapping SFL methods such as GRU and CNN-LSTM are expected to give a better future dynamics prediction with a limited learning time than the linear-mapping SFL methods such as FCN. In line with this reasoning, we are not surprised to observe that the simple linear-mapping TTM approach fails in the triad cases, which is shown in the Supporting Information.

**Figure 7:**
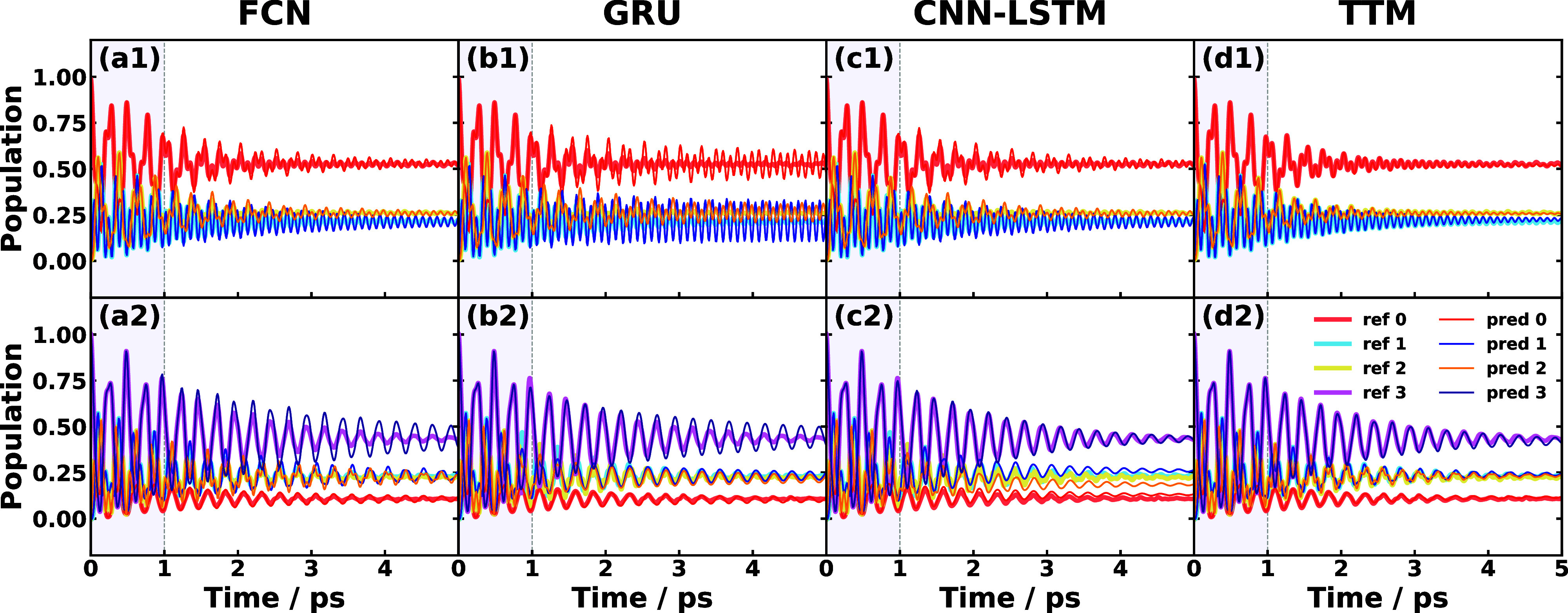
Fixed learning time SFL predictions for the 3-state MSH model #1 (upper) and 4-state MSH model #2 (lower) using different ML approaches including (a) FCN, (b) GRU, (c) CNN-LSTM, and (d) TTM approach. The predicted long-time dynamics of the populations (thin lines) are compared with the reference RI-LSC2 dynamics (thick lines). All the panels use a total learning time of 1 ps, in which the last 0.2 ps is used as hyper-learning time. The effective memory time of MSH #1–2 is estimated to be 0.1 and 1 ps with a tolerance of 10^–5^, respectively.

In Figure 7, we show the fixed-time comparison for the two MSH models with Ohmic spectral densities, of which MSH #1 has three states and MSH #2 has four states. The learning time in both MSH models is restricted to 1 ps, where the last 0.2 ps is used as hyper-learning time. These MSH models have small electronic-vibrational couplings that are derived from the heterogeneous pairwise reorganization energies (typically in 0.25–1.60 cm^–1^), thus underdamped oscillations are expected. In contrast to the previous physical systems, the population dynamics in the nonadiabatic dynamics in the current MSH models with Ohmic spectral densities are perfectly predicted by the TTM method. The CNN-LSTM approach performs the second best, but it overestimates the oscillation amplitude for MSH #1 and produces a slightly shifted equilibrium population for MSH #2. GRU and FCN perform similarly for both MSH models and they overestimate the oscillation amplitude in the population. The overall performance for all SFL ML and TTM methods in the two MSH models is excellent with a fixed learning time of 1 ps, where the linear-mapping TTM is the best among all methods and CNN-LSTM is the best among nonlinear-mapping methods. These systems have a relatively short memory time of around 1 ps, so from our experience in SBMs, the linear-mapping FCN should also perform well, at least as well as TTM. We anticipate that the performance of FCN to increase with a longer learning time and the confirmation of this hypothesis requires a varying learning time test.

**Figure 8:**
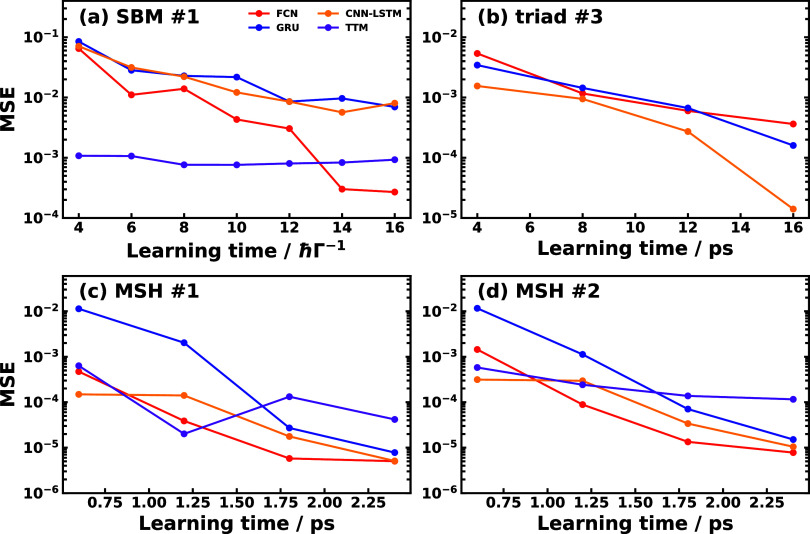
Mean square error (MSE) as a function of learning time for different SFL methods and TTM method in selected physical models: (a) SBM #1, (b) triad conf. 3, (c) MSH #1, and (d) MSH #2. For ML methods, the last 20% of learning time is used as hyper-learning time.

To this end, we show the mean square error (MSE) as a function of learning time in Figure 8 for different SFL methods and TTM methods in the three kinds of physical model including SBM #1, triad conf. 3, and MSH #1 and #2. It is observed that for all three ML methods, the accuracy increases (or the error decreases) with an increasing learning time, while the performance of TTM method does not monotonically depend on the learning time in SBM #1, MSH #1 and #2. It is clear that TTM is not sensitive to the learning time, so TTM could reach a relatively good performance when the learning time is short, but hardly improve the accuracy when increasing the learning time. Indeed, the performance of FCN exceeds the TTM when learning time increases to larger than 1.75 ps in the MSH #1 and #2 models, which confirms our hypothesis that the less accurate prediction with FCN than TTM is due to that the learning time is not long enough to cover the effective memory time and the fact that TTM utilizes more data than FCN. In particular, TTM uses the short-time dynamics starting from *F*^2^ RDM elements while FCN only uses the dynamics starting from a single initial condition. However, the performance of TTM varies and might be influenced by the noise and accuracy in the given learning time data set, and is less robust than the other ML methods. The phenomenon that TTM does not show a clear dependence on the learning time demonstrates the fundamental difference between physics-based transfer tensor and ML-fitted propagator for the RDM.

From the above tests, SBMs and MSH #1 and #2 have short memory time and thus their reduced dynamics can be captured by both linear and nonlinear mapping methods, of which the simple linear-mapping methods such as FCN are less prone to the overfitting issue that might degrade the quality in population prediction using the nonlinear-mapping methods such as CNN-LSTM and GRU with a more complicated network structure. When the memory time is long, for example in the five triad conformations, the nonlinear-mapping methods (CNN-LSTM and GRU) outperform the linear-mapping methods (FCN and TTM), since the more sophisticated neural network structures exhibit better fitting adaptiveness for reduced dynamics that are dissipative. Within linear-mapping methods, FCN is typically better than TTM (when it works), and within nonlinear-mapping methods, CNN-LSTM generally is better than GRU.

**Figure 9:**
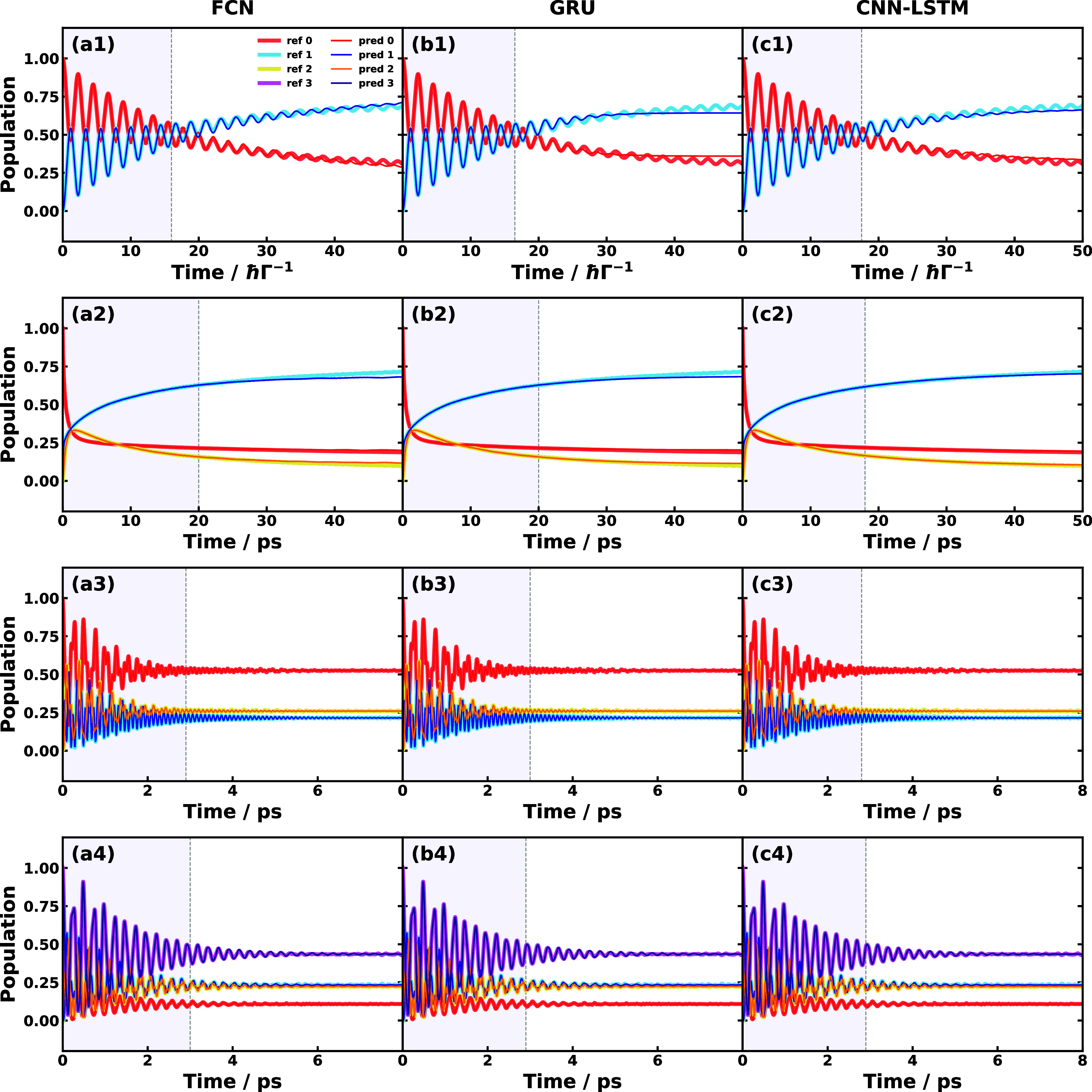
Best performance SFL predictions of populations dynamics using (a) FCN, (b) GRU, (c) CNN-LSTM, and (d) TTM for selected physical models: (1) SBM #1, (2) triad conf. 3, (3) MSH #1, (4) MSH #2. Here, hyper-learning time is not included in the learning time indicated as the shaded area.

Before closing this section, we show the best performance of each SFL ML method could be achieved in all three types of physical models in Figure 9. The learning time length is now treated as a hyperparameter that is optimized to minimize the cost function and the allowed learning time is up to 20 *ℏ* Γ^–1^ in SBM #1, up to 20 ps in triad conf. 3, and up to 3 ps in MSH #1 and #2. All the SFL ML methods perform well and almost coincide with the reference dynamics, except for the cases of overdamped prediction of GRU and CNN-LSTM for SBM #1 and the cases of slight derivations seen in FCN and GRU predictions for triad conf. 3. In practice, using such a long learning time is not preferred since that will diminish the benefit of applying the SFL ML methods to save computational resources for generating long-time dynamics. The best performance test indicates that the propagation of the reduced dynamics can be captured by both linear and nonlinear mapping ML methods if long enough learning time input is provided, and numerically demonstrates the linear-mapping formulation of non-Markovian dynamics with long enough effective memory time described in Sec. 2. It is also worth mentioning that the ML approaches are expected to reproduce the future dynamics given short-term dynamics as input faithfully, and the accuracy should be evaluated by the deviation from the result generated by the same dynamical method even if there is a systematic error of this particular dynamical method. Unlike the case when using approximate input dynamics in GQME, from the ML perspective, it does not mean an ML model is an impressive one, even though the predicted long-time dynamics are closer to the exact quantum dynamics than the original quasiclassical dynamics.

## 6 Concluding Remarks

In this work, we systematically test the performance of three time-series ML methods and the physics-based transfer tensor method for predicting the long-time nonadiabatic dynamics given short-time reduced dynamics as input. The SFL ML methods include FCN, GRU, and CNN-LSTM approaches, of which FCN features linear-mapping propagation and the rest feature nonlinear-mapping propagation. From the formally exact Nakajima–Zwanzig generalized quantum master equation, we show that the non-Markovian dynamics can be represented as a linear map when the initial density matrix is a product state of system and bath, as well as the knowledge of the historical dynamics is long enough to cover the memory time. The requirement of historical knowledge in non-Markovian dynamics of reduced density matrix originates from the ignorance of the environment status of open quantum systems. However, in practice, it is not always straightforward to estimate how long one needs to know the history of reduced information to predict the future. We proposed an effective memory time indicator, which could give practical guidance on choosing the truncating time, which is expected to give rise to a propagation error less than a certain threshold. From the distribution of the indicator on different tolerance levels, one could determine the effective memory time for SFL ML and TTM methods.

We applied the three SFL ML and TTM methods in different physical systems, including two-electronic-state spin-boson models, three-state MSH Hamiltonian for five conformations of the carotenoid-porphyrin-fullerene triad in solution, and three-state and four-state MSH Hamiltonians with Ohmic spectral densities. The loss function is regularized with the total population restriction, which is used in training and hyper-training. Results suggest that different physical models have rather distinguished non-Markovian effects: linear-mapping FCN works better for systems with short memory time and nonlinear-mapping GRU and CNN-LSTM work better for systems with long memory time. First, the spin-boson models and the MSH Hamiltonians with Ohmic spectral densities have short memory time, and thus their population dynamics can be captured by both linear-mapping FCN and nonlinear-mapping GRU and CNN-LSTM methods (CNN-LSTM is typically better than GRU). In particular, the linear-mapping FCN gives rise to the best performance for the above-mentioned physical models compared with nonlinear-mapping GRU and CNN-LSTM, which may suffer from overfitting problems. Second, the five triad conformations dissolved in organic solvent at 300 K have a relatively long memory time, which can be captured with nonlinear-mapping GRU and CNN-LSTM since they have sophisticated neural network structures. Third, the linear-mapping TTM is sensitive to the noise and accuracy in the dynamics input but insensitive to learning time, which leads to unique applicability in SFL prediction. Thus, this work demonstrates different SFL methods are suitable for various physical systems and the effective memory time indicator technique could provide practical guidance. An astonishing discovery here is the best performance in predicting nonadiabatic dynamics in systems with short memory times can be achieved by the simplest FCN. We believe the strategy described here would be applicable for many more occasions for predicting long-time dynamics, such as using other state-of-the-art time-series ML methods such as transformer with the attention mechanism^75,76^ and the derived temporal fusion transformer (TFT)^77^ and inverted transformer,^78^ as well as the structured state space models (SSMs)-based Mamba,^79,80^ which have been developed to tackle the computational inefficiency of transformer when processing long sequences. Additionally, there are MTGNN based on graph neural networks (GNN)^81^ and TimeMixer based on multiple layer projection (MLP).^82^ Work on these directions is underway and will be reported in future publications.

## Supporting Information Available

Description of the transfer tensor method, long short-term memory method, spin-boson model parameters, MSH model parameters, realistic triad spectral densities, effective time indicators in different systems, time step size tests, TTM result for five triad conformations, SFL results for Fenna-Metthews-Olson models and linear vibronic coupling models, FCN with nonlinear activation functions, and the SFL performance test for different learning times (PDF)
