# Supplementary material for: How Sophisticated Are Neural Networks Needed to Predict Long-Term Nonadiabatic Dynamics?
Source: J Chem Theory Comput. 2024 Nov 14;20(22):9832–48. doi: 10.1021/acs.jctc.4c01223 (PMC11603613; doi:10.1021/acs.jctc.4c01223)
Supplement: Supplementary file 1 — ct4c01223_si_001.pdf [file ct4c01223_si_001.pdf]

Supporting Information:
How Sophisticated Are Neural Networks Needed to Predict Long-Term Nonadiabatic Dynamics?

Hao Zeng,[1,2,3,4] Yitian Kou,[1,5,3] and Xiang Sun[3,1,4,2,6,a)]
[1)] *Shanghai Frontiers Science Center of Artificial Intelligence and Deep Learning, NYU Shanghai, 567 West Yangsi Road, Shanghai, 200124, China*
[2)] *State Key Laboratory of Precision Spectroscopy, East China Normal University, Shanghai 200062, China*
[3)] *Division of Arts and Sciences, NYU Shanghai, 567 West Yangsi Road, Shanghai 200124, China*
[4)] *NYU-ECNU Center for Computational Chemistry at NYU Shanghai, 3663 Zhongshan Road North, Shanghai 200062, China*
[5)] *School of Computer Science and Technology, East China Normal University, Shanghai 200062, China*
[6)] *Department of Chemistry, New York University, New York, New York 10003, United States*

I. TRANSFER TENSOR METHOD (TTM)

For completeness, we briefly summarize the transfer tensor method (TTM) proposed by Cerrillo and Cao.[1] TTM is a kind of black-box technique based on a dynamical map $\mathcal{E}_n$, which is a superoperator that maps the initial density matrix to the density matrix at time t_n:

$$\hat{\rho}(t_n) = \mathcal{E}_n \hat{\rho}(0), \tag{S1}$$

where $t_n = n\Delta t$ and Δt is time step of simulation. Then we can transform the dynamical map into the following form

$$\mathcal{T}_{n,0} = \mathcal{E}_n - \sum_{k=0}^{n-1} \mathcal{T}_{n,k}\mathcal{E}_k, \tag{S2}$$

where $\mathcal{T}_{n,k}$ is the transfer tensor. To this end, the dynamical propagation of density matrix can be rewritten as

$$\hat{\rho}(t_n) = \sum_{k=0}^{n-1} \mathcal{T}_{n,k}\hat{\rho}(t_k). \tag{S3}$$

Suppose the dynamical maps are linear, we can construct the elements of dynamical maps $\mathcal{E}_{n,jklm}$ from known dynamics:

$$\mathcal{E}_{n,jklm} \equiv \mathrm{Tr}\left\{(|j\rangle\langle k|)^{\dagger}\mathcal{E}_n|l\rangle\langle m|\right\} = \mathrm{Tr}\left\{|k\rangle\langle j|\hat{\rho}_{lm}(t_n)\right\}. \tag{S4}$$

Here, $\{|j\rangle\}$ are the basis, and $\hat{\rho}_{lm}(t_n)$ is the density matrix at time t_n with $|l\rangle\langle m|$ as initial condition. We can obtain the transfer tensors iteratively with Eq. S2, and propagate the dynamics with Eq. S3.

II. LONG SHORT-TERM MEMORY (LSTM)

Long short-term memory (LSTM) and gated recurrent unit (GRU) are two typical types of Recurrent Neural Networks (RNNs) designed to solve the vanishing gradient problem faced by traditional RNNs. They both use gating mechanisms to control the flow of information, which helps to decide which information to retain or discard, allowing them to capture long-term dependencies and mitigate the problem of vanishing gradients.

As shown in Fig. S1, LSTM has three gates: input gate, forget gate, and output gate. The input gate controls how much new information flows into the cell state, the forget gate decides what information to discard, and the output gate determines the output based on the cell state. At the first time step of the sequence, the hidden state h_0 and the cell state c_0 are initialized as zero vectors. Specifically, the computations within an LSTM cell for a time step t are as follows:

[a)] Electronic mail: xiang.sun@nyu.edu

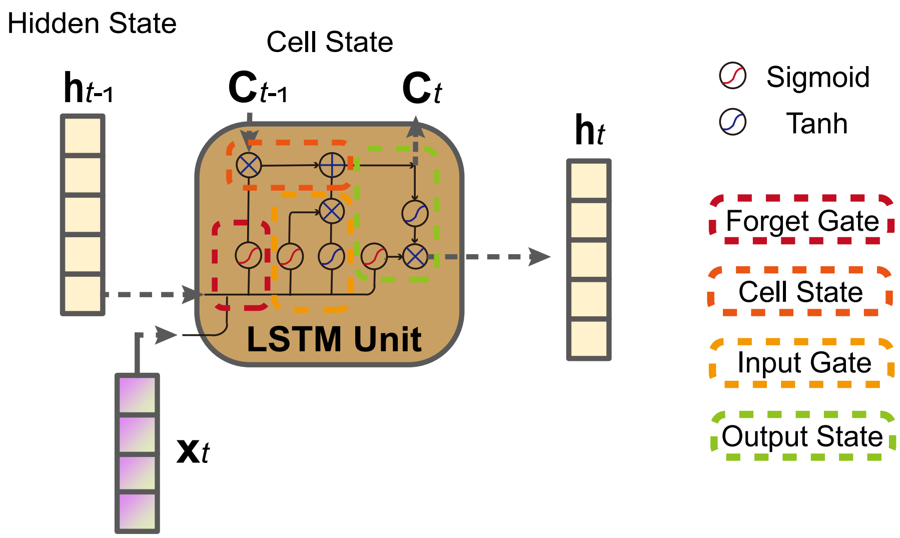

FIG. S1. Schematic illustration of the inner structure of an LSTM unit.

1. Forget gate $(\mathbf{f}_t)$

   The forget gate decides which information to discard from the cell state. It takes the current input $\mathbf{x}_t$ and the previous hidden state $\mathbf{h}_{t-1}$ and applies a sigmoid activation function:

$$\mathbf{f}_t = \text{sig}\left(\mathbf{W}_f\left[\mathbf{h}_{t-1}, \mathbf{x}_t\right] + \mathbf{b}_f\right), \tag{S5}$$

   where $\mathbf{W}_f$ is the weight matrix for the forget gate, $\mathbf{b}_f$ is the bias term for the forget gate, and $\text{sig}(x)$ is the sigmoid activation function $\text{sig}(x) = 1/(1+e^{-x})$.

2. Input gate $(\mathbf{i}_t)$

   The input gate decides which new information to add to the cell state. It also uses the sigmoid activation function:

$$\mathbf{i}_t = \text{sig}\left(\mathbf{W}_i \cdot \left[\mathbf{h}_{t-1}, \mathbf{x}_t\right] + \mathbf{b}_i\right), \tag{S6}$$

   where $\mathbf{W}_i$ is the weight matrix for the input gate and $\mathbf{b}_i$ is the bias term for the input gate.

3. Candidate cell state $\left(\tilde{\mathbf{C}}_t\right)$

   The candidate cell state is a new value created using the tanh activation function, representing new potential information to be added to the cell state:

$$\tilde{\mathbf{C}}_t = \tanh\left(\mathbf{W}_C \cdot \left[\mathbf{h}_{t-1}, \mathbf{x}_t\right] + \mathbf{b}_C\right), \tag{S7}$$

   where $\mathbf{W}_C$ is the weight matrix for the candidate cell state, $\mathbf{b}_C$ is the bias term for the candidate cell state, tanh is the hyperbolic tangent activation function $\tanh(x) = \frac{e^x - e^{-x}}{e^x + e^{-x}}$.

4. Update the cell state $(\mathbf{C}_t)$

   The cell state is updated by combining the old cell state $(\mathbf{C}_{t-1})$ and the new candidate cell state ($\tilde{\mathbf{C}}_t$) using the forget gate and input gate:

$$\mathbf{C}_t = \mathbf{f}_t \odot \mathbf{C}_{t-1} + \mathbf{i}_t \odot \tilde{\mathbf{C}}_t, \tag{S8}$$

   where $\odot$ denotes element-wise multiplication.

5. Output gate $(\mathbf{o}_t)$

   The output gate determines the output based on the updated cell state. It uses the sigmoid activation function:

$$\mathbf{o}_t = \text{sig}\left(\mathbf{W}_o \cdot \left[\mathbf{h}_{t-1}, \mathbf{x}_t\right] + \mathbf{b}_o\right), \tag{S9}$$

   where $\mathbf{W}_o$ is the weight matrix for the output gate, $\mathbf{b}_o$ is the bias term for the output gate.

6. Compute the hidden state ($\mathbf{h}_t$)

   The hidden state is the final output of the LSTM cell. It is computed by applying the output gate to the tanh of the updated cell state:

$$\mathbf{h}_t = \mathbf{o}_t \odot \tanh(\mathbf{C}_t). \tag{S10}$$

Comparison between LSTM and GRU

GRU has two gates: an update gate and a reset gate. The update gate controls how much of the past information should be carried forward to the future, and the reset gate determines how much of the past information should be forgotten. Besides, GRU combines the cell state and the hidden state into a single hidden state, making it simpler in architecture and reducing the number of parameters. GRU often performs similarly to LSTM but has fewer parameters, making it more computationally efficient and faster to train.

III. SPIN-BOSON MODEL

The frequencies $\{\omega_j\}$ and the electronic-vibrational coupling coefficients $\{c_j\}$ of the spin-boson models are determined by discretizing the Ohmic spectral density:

$$J(\omega) = \frac{\pi}{2}\sum_{j=1}^{N}\frac{c_j^2}{\omega_j}\delta(\omega-\omega_j) \xrightarrow{N\to\infty} \frac{\pi}{2}\hbar\xi\omega e^{-\omega/\omega_c} \tag{S11}$$

where ξ is the Kondo parameter and ω_c is the cutoff frequency. The discretization strategy for the Ohmic spectral density is given by

$$\omega_j = -\omega_c \ln\left(1 - j\frac{\omega_0}{\omega_c}\right), \qquad c_j = \sqrt{\xi\hbar\omega_0\omega_j}. \tag{S12}$$

Here, $\omega_0 = \frac{\omega_c}{N}\left(1-e^{-\omega_{\max}/\omega_c}\right)$, where $\omega_{\max}$ is the highest frequency.

TABLE S1. Spin-boson model parameters given in the reduced unit.

Model No.	ε	Γ	E_r	ξ	ω_c	$\omega_{\max}$	N
1	1.0	1.0	0.198	0.1	1.0	5.0	60
2	1.0	1.0	0.397	0.1	2.0	10.0	60
3	1.0	1.0	1.488	0.1	7.5	36.0	60
4	0	1.0	0.992	0.2	2.5	12.0	60

The initial nuclear sampling of spin-boson models is performed at $\beta = 1/k_BT = 5.0$.

IV. MSH MODEL

To construct the equilibrium shift components in the MSH model, we need the molecular inputs in terms of the energy-gap time correlation functions (TCFs) between all pairs of states from the all-atom molecular dynamics (MD) simulations. In the F-state case, there are $F(F-1)/2$ energy-gap TCFs $\{C_{UU}^{(XY)}(t)|X<Y, \text{ and } X,Y\in 1,\ldots,F\}$. From these TCFs, we can obtain the corresponding reorganization energies between any pair of states:

$$E_r^{(XY)} = \frac{C_{UU}^{(XY)}(0)}{2k_BT}. \tag{S13}$$

The N normal-mode frequencies $\{\omega_i\}$ can be then obtained by solving the following equation:

$$\frac{2N\omega_i}{\pi C_{UU}^{(XY)}(0)}\int_0^\infty \mathrm{d}t \frac{C_{UU}^{(XY)}(t)}{\omega_i t}\sin(\omega_i t) = i - \frac{1}{2}, \tag{S14}$$

and the equilibrium geometry distances between the PES minima of X,Y states, $R_i^{(XY)}$, are given by

$$R_i^{(XY)} = \sqrt{\frac{2E_r^{(XY)}}{N}}\frac{1}{\omega_i},\ \ (i=1,\ldots,N) \tag{S15}$$

which are related to the electronic-nuclear couplings. The reorganization energy can also be written as

$$E_r^{(XY)} = \sum_{i=1}^{N}\frac{1}{2}\omega_i^2(R_i^{(XY)})^2. \tag{S16}$$

Thus, there are $F(F-1)/2$ reorganization energies corresponding to $F(F-1)/2$ pairs of electronic states, which are directly obtained from all-atom MD simulations. The realistic spectral densities of the five triad conformations are shown in Fig. S2. The equilibrium distance between any two states is proportional to the square root of the associated reorganization energy (see Eq. S15). To satisfy all reorganization energy constraints in the model Hamiltonian, one needs to extend to $F-1$ dimensions for each physical normal mode.[2] As demonstrated in Ref. 3, the MSH model faithfully reproduces the nonadiabatic dynamics of the triad in explicit THF solvent. The $F(F-1)/2$ sets of equilibrium shift components $\{S_i^{(aX)}|a<X\}$ simultaneously satisfy the $F(F-1)/2$ reorganization energy constraints. This means that $\{S_i^{(aX)}\}$ should ensure the distance between the minima of all pairs of PESs scales linearly with the square root of the corresponding reorganization energy, as shown in Eq. S15. The resulting equilibrium shift components are thus given by[2]

$$\begin{pmatrix} S_i^{(12)} & & \\ S_i^{(13)} & S_i^{(23)} & \\ S_i^{(14)} & S_i^{(24)} & S_i^{(34)} \\ & \cdots & \end{pmatrix} = \sqrt{\frac{2}{N}}\frac{1}{\omega_i}\begin{pmatrix} A_1^{(12)} & & \\ A_1^{(13)} & A_2^{(13)} & \\ A_1^{(14)} & A_2^{(14)} & A_3^{(14)} \\ & \cdots & \end{pmatrix}, \tag{S17}$$

where the PES minima in $(F-1)$-dimensional extended space are

$$\begin{aligned}
A^{(12)} &= \sqrt{E_r^{(12)}}(1,0,\ldots,0),\\
A^{(13)} &= \sqrt{E_r^{(13)}}(\cos\theta_{23},\sin\theta_{23},0,\ldots,0),\\
A^{(14)} &= \sqrt{E_r^{(14)}}(\cos\theta_{24},\sin\theta_{24}\cos\theta'_{34},\sin\theta_{24}\sin\theta'_{34},0,\ldots,0),\\
&\cdots\\
A^{(1F)} &= \sqrt{E_r^{(1F)}}\big(\cos\theta_{2F},\sin\theta_{2F}\cos\theta'_{3F},\sin\theta_{2F}\sin\theta'_{3F}\cos\theta'_{4F},\\
&\sin\theta_{2F}\sin\theta'_{3F}\sin\theta'_{4F}\cos\theta'_{5F},\sin\theta_{2F}\sin\theta'_{3F}\sin\theta'_{4F}\sin\theta'_{5F}\cos\theta'_{6F},\\
&\ldots,\\
&\sin\theta_{2F}\sin\theta'_{3F}\sin\theta'_{4F}\sin\theta'_{5F}\cdots\sin\theta'_{F-2,F}\cos\theta'_{F-1,F},\\
&\sin\theta_{2F}\sin\theta'_{3F}\sin\theta'_{4F}\sin\theta'_{5F}\cdots\sin\theta'_{F-2,F}\sin\theta'_{F-1,F}\big),
\end{aligned} \tag{S18}$$

where

$$\cos\theta_{jk} = \frac{E_r^{(1j)}+E_r^{(1k)}-E_r^{(jk)}}{2\sqrt{E_r^{(1j)}E_r^{(1k)}}}, \tag{S19}$$

$$\cos\theta'_{3k} = \frac{\cos\theta_{3k}-\cos\theta_{23}\cos\theta_{2k}}{\sin\theta_{23}\sin\theta_{2k}},\ (k\geq 4), \tag{S20}$$

and

$$\begin{aligned}
\cos\theta'_{jk} = \Big[&\cos\theta_{jk}-\cos\theta_{2j}\cos\theta_{2k}-\sin\theta_{2j}\sin\theta_{2k}\cos\theta'_{3j}\cos\theta'_{3k}-\cdots\\
&-\sin\theta_{2j}\sin\theta_{2k}\sin\theta'_{3j}\sin\theta'_{3k}\sin\theta'_{4j}\sin\theta'_{4k}\cdots\sin\theta'_{(j-2)j}\sin\theta'_{(j-2)k}\cos\theta'_{(j-1)j}\cos\theta'_{(j-1)k}\Big]\\
&\times\Big[\sin\theta_{2j}\sin\theta_{2k}\sin\theta'_{3j}\sin\theta'_{3k}\sin\theta'_{4j}\sin\theta'_{4k}\cdots\sin\theta'_{(j-2)j}\sin\theta'_{(j-2)k}\sin\theta'_{(j-1)j}\sin\theta'_{(j-1)k}\Big]^{-1},\ (4\leq j<k).
\end{aligned} \tag{S21}$$

The initial nuclear sampling in all seven MSH models is performed at 300 K.

TABLE S2. The system Hamiltonians of triad conf. 1-5 MSH models, energy in eV.

	conf. 1	conf. 2	conf. 3	conf. 4	conf. 5
ε_0	0	0	0	0	0
ε_1	-0.952	-0.268	-0.828	-0.156	-0.758
ε_2	-1.080	-0.779	-0.640	-0.669	-1.128
ε_3	0	0	0	0	0
Γ_{01}	2.408×10^{-2}	8.810×10^{-2}	-1.467×10^{-2}	-6.588×10^{-2}	8.104×10^{-2}
Γ_{02}	4.472×10^{-5}	1.149×10^{-4}	7.201×10^{-3}	8.431×10^{-4}	4.102×10^{-3}
Γ_{03}	0	0	0	0	0
Γ_{12}	8.602×10^{-5}	1.185×10^{-4}	-2.924×10^{-2}	-7.662×10^{-4}	-3.242×10^{-3}
Γ_{13}	0	0	0	0	0
Γ_{23}	0	0	0	0	0
$E_r^{(01)}$	0.509	0.311	0.340	0.177	0.280
$E_r^{(02)}$	1.462	1.507	0.492	0.773	0.810
$E_r^{(03)}$	0.090	0.127	0.150	0.043	0.013
$E_r^{(12)}$	1.418	1.316	0.041	0.256	0.877
$E_r^{(13)}$	0.905	0.559	0.910	0.328	0.343
$E_r^{(23)}$	1.696	1.699	1.134	1.076	0.822

TABLE S3. The system Hamiltonians of MSH #1 and #2 models, energy in cm^{-1}.

	MSH #1	MSH #2
ε_0	100	-100
ε_1	-100	0
ε_2	0	50
ε_3	/	150
Γ_{01}	100	100
Γ_{02}	10	100
Γ_{03}	/	0
Γ_{12}	100	100
Γ_{13}	/	0
Γ_{23}	/	100
$E_r^{(01)}$	0.252	1.000
$E_r^{(02)}$	0.500	1.366
$E_r^{(03)}$	/	1.605
$E_r^{(12)}$	0.683	0.952
$E_r^{(13)}$	/	1.191
$E_r^{(23)}$	/	0.971

V. EFFECTIVE MEMORY TIME INDICATORS

We have shown the effective memory time indicators $I_\varepsilon^M(K)$ with different M and different tolerances ε for MSH #1 model in the main text. Here, we show the indicators at different tolerances ε for the other models – SBM #1, triad conf. 3, and MSH #2 – to see what the distributions of indicators are in these models, and how the distributions relate to the sufficiency of truncating time. From Fig. S3(a), we can see the indicator curve spread widely on the horizontal axis, which means the effective memory time should be covered within the max time of $5\hbar\Gamma^{-1}$. For example, we can determine the effective memory time of $1.2\hbar\Gamma^{-1}$ with a tolerance $\varepsilon = 5\times10^{-5}$. Next, Fig. S3 (b) shows the indicators for the triad conf. 3 model, where all the curves distribute heavily at the end of the horizontal axis (5 ps), even when the tolerance is set to such a large value of 1×10^{-2}. This means that the non-Markovian propagators do not vanish at the time of 5 ps, thus the methods based on the linear transformation are expected to fail in this long-memory model. Thus, we are not surprised to observe that TTM fails to predict the correct dynamics in all triad conf. 1-5 models featuring long memories, as shown in Fig. S4. Moreover, Fig. S3(c) shows indicators for MSH #2, where the indicator at $\varepsilon = 1\times10^{-4}$ determines an effective truncating time of about 0.5 ps reflecting that MSH #2 is a short-memory system.

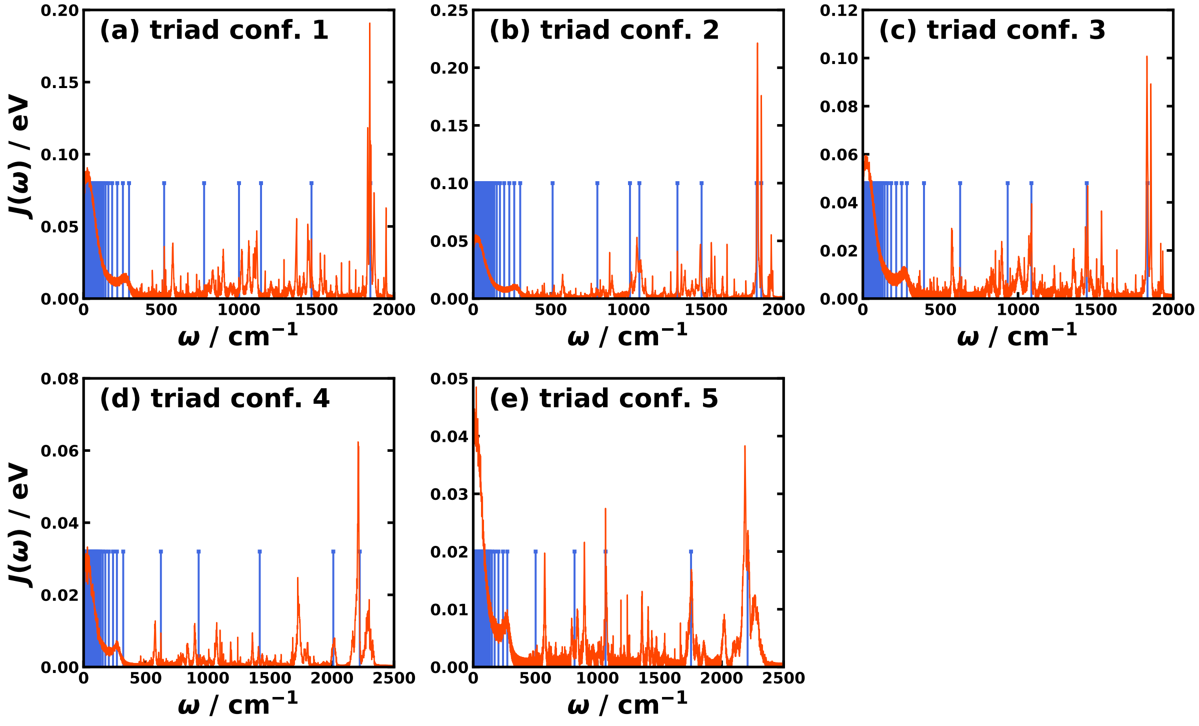

FIG. S2. The realistic spectral densities of triad conf. 1–5 (red curve) and the corresponding discretized frequencies (blue bars).

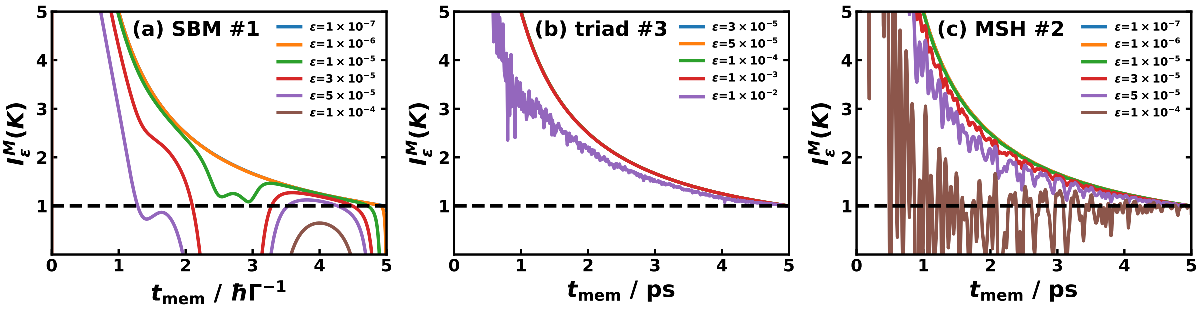

FIG. S3. Indicators $I_\varepsilon^M(K)$ at truncating time t_{mem} for (a) SBM #1 (b) triad conf. 3 (c) MSH #2 of different tolerances ε with fixed max time of 5 $\hbar\Gamma^{-1}$/ps.

VI. RESULTS FOR FENNA–MATTHEWS–OLSON (FMO), LINEAR VIBRONIC COUPLING (LVC) MODEL, AND LVC GARG-ONUCHIC-AMBEGAOKAR (GOA) MODELS

Besides the models reported in the main text, we also test the methods on four-state FMO of *C. tepidum*, two-state LVC model for 2-methylene-6isopropylidene adamantyl (MIA) radical cation with a conical intersection, and two-state LVC GOA model. The parameters of LVC MIA and GOA can be found in Refs. 4,5. The FMO model is described within the MSH framework with orthogonal equilibrium shift vectors, whose detailed parameters are listed in Table S4. The initial nuclear sampling of FMO and LVC MIA is performed at temperature 300 K and the GOA model is done at a temperature of $\beta = 1/k_BT = 5.0$.

The results are shown in the Fig. S5. TTM can predict the FMO and MIA model well with carefully chosen learning time, which is very tricky to decide since the non-Markovian propagators do not decay to zero but increase after a short time. Thus, it is also not straightforward to select this effective learning time for the specific models, if the future dynamics are not known. TTM performs badly when using a coherence element as the initial reduced density matrix. Most ML methods fail in these three models, except for the cases where GRU and CNN-LSTM perform well for the MIA model. It seems to indicate that the semiclassical dynamical methods might not produce the exact quantum dynamics within the memory time, and the noise and deviation from the exact dynamics prevent these ML methods from fitting the exact non-Markovian propagators. Further

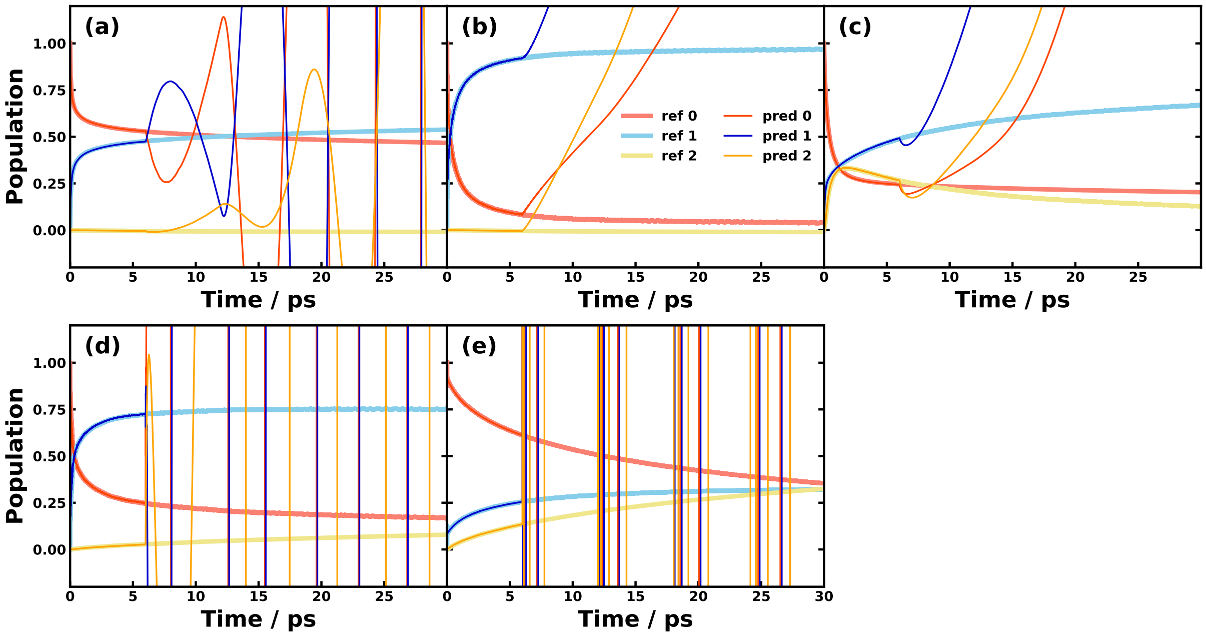

FIG. S4. Prediction results of TTM for (a) triad conf. 1, (b) triad conf. 2, (c) triad conf. 3, (d) triad conf. 4 and (e) triad conf. 5 with fixed learning time of 6 ps.

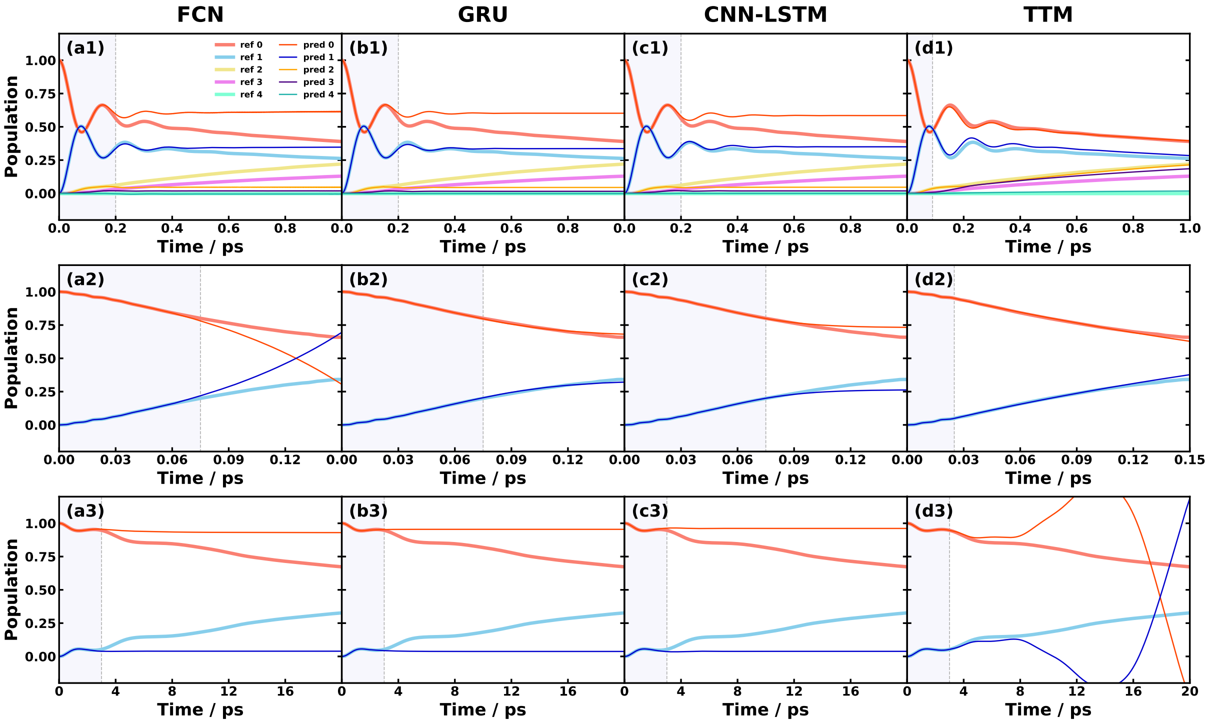

FIG. S5. SFL predictions for FMO, MIA and GOA model using different ML approaches including (a) FCN, (b) GRU, (c) CNN-LSTM, and (d) TTM approach. The three rows correspond to (1) FMO (2) MIA and (3) GOA. The predicted long-time dynamics of the populations (thin lines) are compared with the reference RI-LSC2 dynamics (thick lines). The total learning times of ML and TTM methods are marked with the right edge of the grey boxes.

comparison of these models with numerically exact results may be required to reveal the origin of the failure in predicting the long-term dynamics of FMO, MIA, and GOA models.

	FMO
ε_0	12410
ε_1	12530
ε_2	12210
ε_3	12320
ε_4	0
Γ_{01}	-87.7
Γ_{02}	5.5
Γ_{03}	-5.9
Γ_{04}	0
Γ_{12}	30.8
Γ_{13}	8.2
Γ_{14}	0
Γ_{23}	-53.5
Γ_{24}	0
Γ_{34}	0
$E_r^{(01)}$	92.511
$E_r^{(02)}$	92.880
$E_r^{(03)}$	73.738
$E_r^{(04)}$	34.654
$E_r^{(12)}$	113.900
$E_r^{(13)}$	97.806
$E_r^{(14)}$	57.422
$E_r^{(23)}$	95.850
$E_r^{(24)}$	57.906
$E_r^{(34)}$	39.284

TABLE S4. The system Hamiltonian of 4-state FMO model, energy in cm^{-1}.

VII. TESTS FOR NONLINEAR ACTIVATION FUNCTIONS IN FULLY-CONNECTED NEURAL NETWORK (FCN)

We reported the linear FCN with no hidden layer and activation function in the main text. Here, we show the tests for the nonlinear activation functions in FCN in Fig. S6, which are worse than the linear FCN. The performance of FCN with nonlinear activation functions including sigmoid and ReLU is similar to the nonlinear ML methods (GRU and CNN-LSTM): overdamping is observed in the spin-boson model, and the performance for triad conf. 3 is still worse than CNN-LSTM. Thus, for the physical models studied in this work, linear FCN performs better than nonlinear FCN.

VIII. TIME STEP SIZE TEST

We test the different time step sizes in short-memory systems, including SBM #1, MSH #1, and MSH #2 using TTM and ANN, as well as long-memory triad conf. 3 using CNN-LSTM and GRU. The comparison of different time step sizes Δt are shown in Fig. S7. In panels (b1)-(b3), we observe that physics-based TTM that does not rely on fitting is insensitive to Δt in the parameter region we tested. However, the situation for those ML methods that require fitting or optimization of parameters is more complicated than TTM. For some model systems such as MSH #1, MSH #2, and triad conf. 3, change of Δt only leads to a negligible difference in the SFL prediction, while the results differ more in SBM #1. The deviation might originate from two sources: (1) the discretization of time steps and (2) the instability of the ML methods due to the optimization of parameters arriving at local minima rather than the global minima if insufficient dataset is provided. For the time being, we do not have an effective way to distinguish the two sources of deviation in the population dynamics prediction of ML methods.

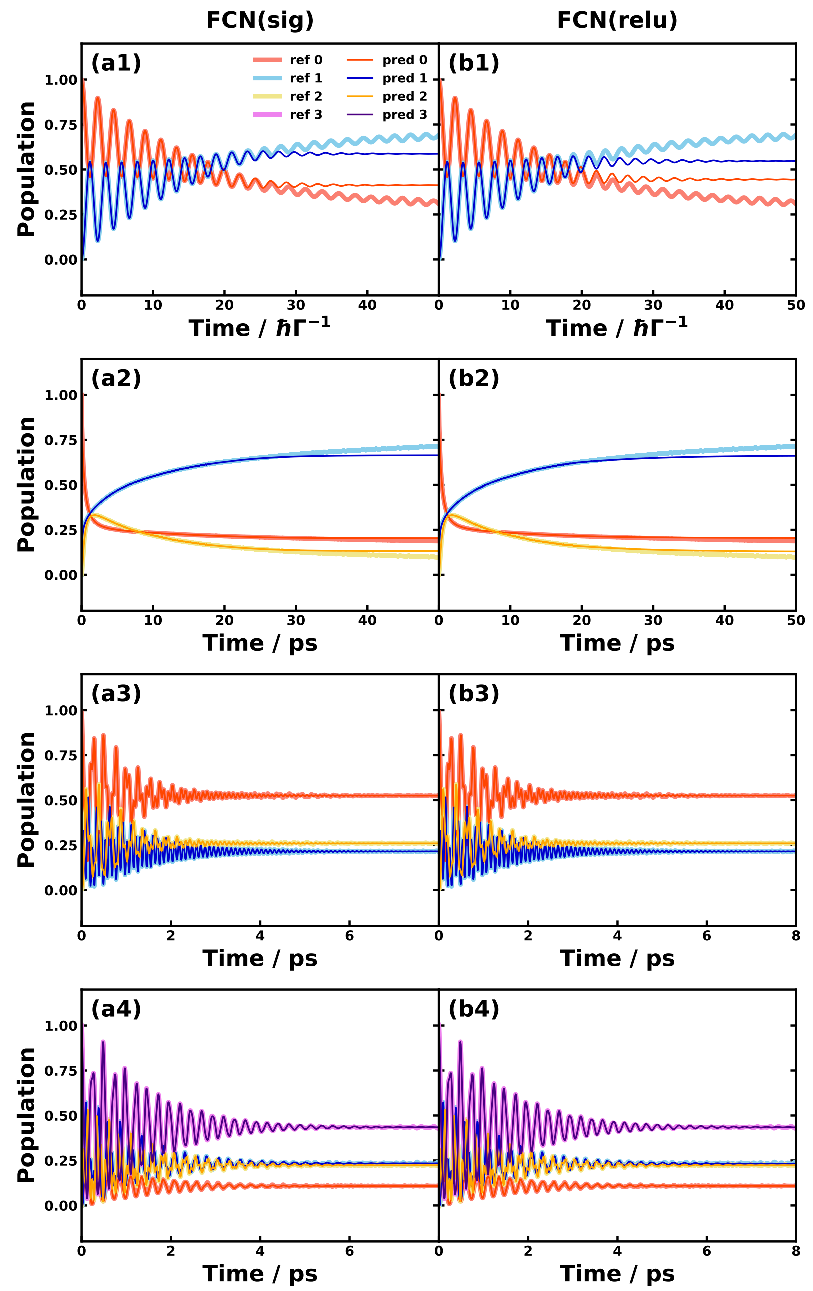

FIG. S6. Best performance SFL predictions using two different nonlinear FCN: (a) 3-hidden-layer FCN with sigmoid activation function, (b) 3-hidden-layer FCN with ReLU activation function for selected physical models: (1) SBM #1, (2) triad conf. 3, (3) MSH #1, (4) MSH #2. The predicted long-time dynamics of the populations (thin lines) are compared with the reference RI-LSC2 dynamics (thick lines). Here, hyper-learning time is not included in the learning time indicated as the shaded area.

IX. LEARNING TIME TEST

We have shown the MSE vs. learning time plot in the main text. Here, we show the results of the learning-time test in population dynamics prediction in Fig. S8, S9, S10, and S11 for SBM #1, triad conf. 3, MSH #1 and #2, respectively. The trend can be seen clearly that the performance of the ML methods improves as learning time increases, but the overfitting issue prevents the nonlinear methods from improving accuracy for short-memory models with strong oscillation. Besides, TTM is not sensitive to learning time once it has covered the memory time.

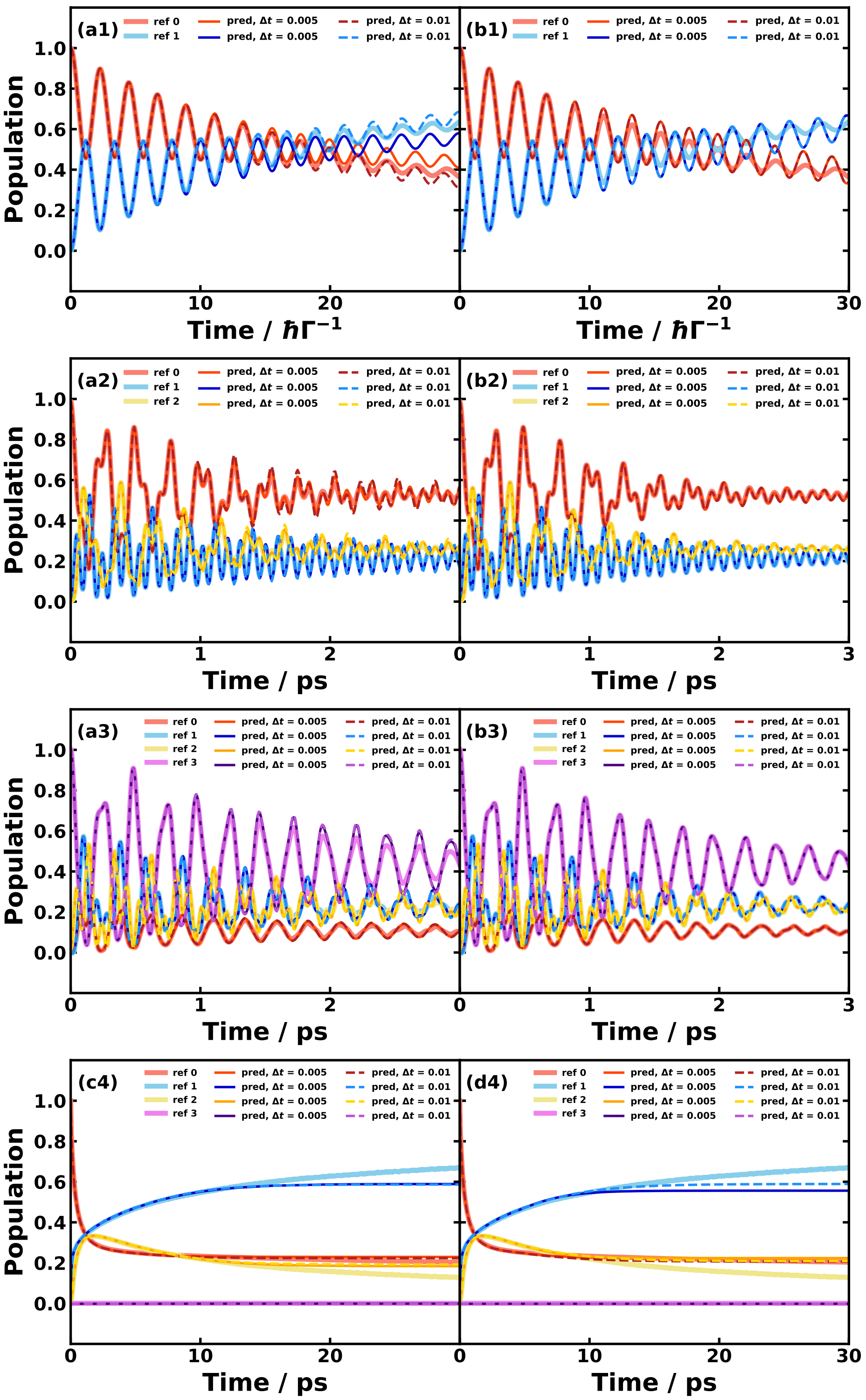

FIG. S7. SFL predictions for (1) SBM #1, (2) MSH #1, (3) MSH #2 and (4) triad conf. 3 models of different SFL methods: (a) FCN, (b) TTM, (c) CNN-LSTM, and (d) GRU with different time step sizes. The predicted long-time dynamics of the populations (thin lines) are compared with the reference RI-LSC2 dynamics (thick lines). Here, hyper-learning time is the last 20% of the learning time, which is indicated as the shaded area.

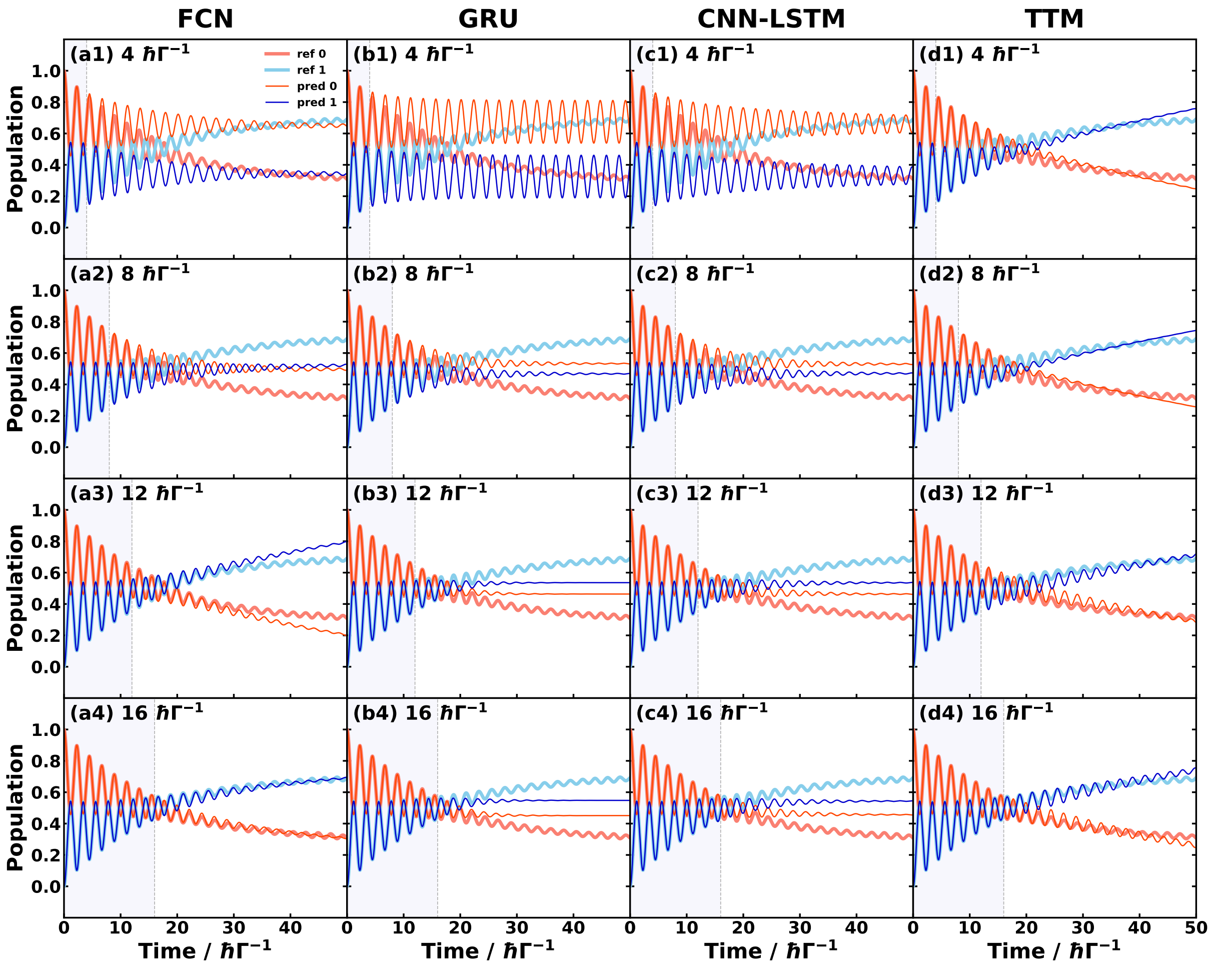

FIG. S8. SFL predictions for SBM #1 using different methods: (a) FCN, (b) GRU, (c) CNN-LSTM, and (d) TTM. The predicted long-time dynamics of the populations (thin lines) are compared with the reference RI-LSC2 dynamics (thick lines). Here, hyper-learning time is the last 20% of the learning time, which is indicated as the shaded area.

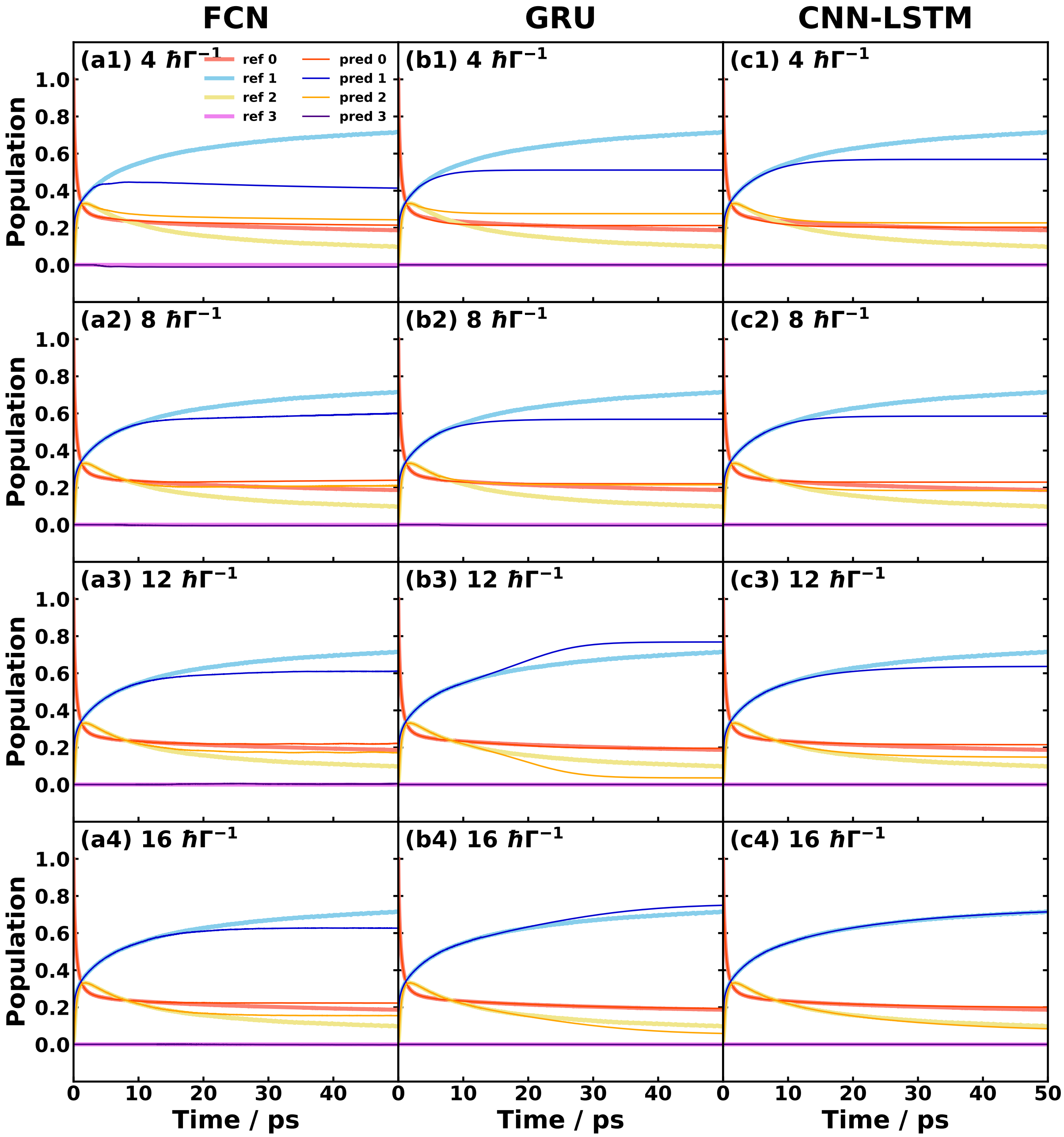

FIG. S9. SFL predictions for triad conf. 3 using different methods: (a) FCN, (b) GRU, (c) CNN-LSTM. The predicted long-time dynamics of the populations (thin lines) are compared with the reference RI-LSC2 dynamics (thick lines). Here, hyper-learning time is the last 20% of the learning time, which is indicated as the shaded area.

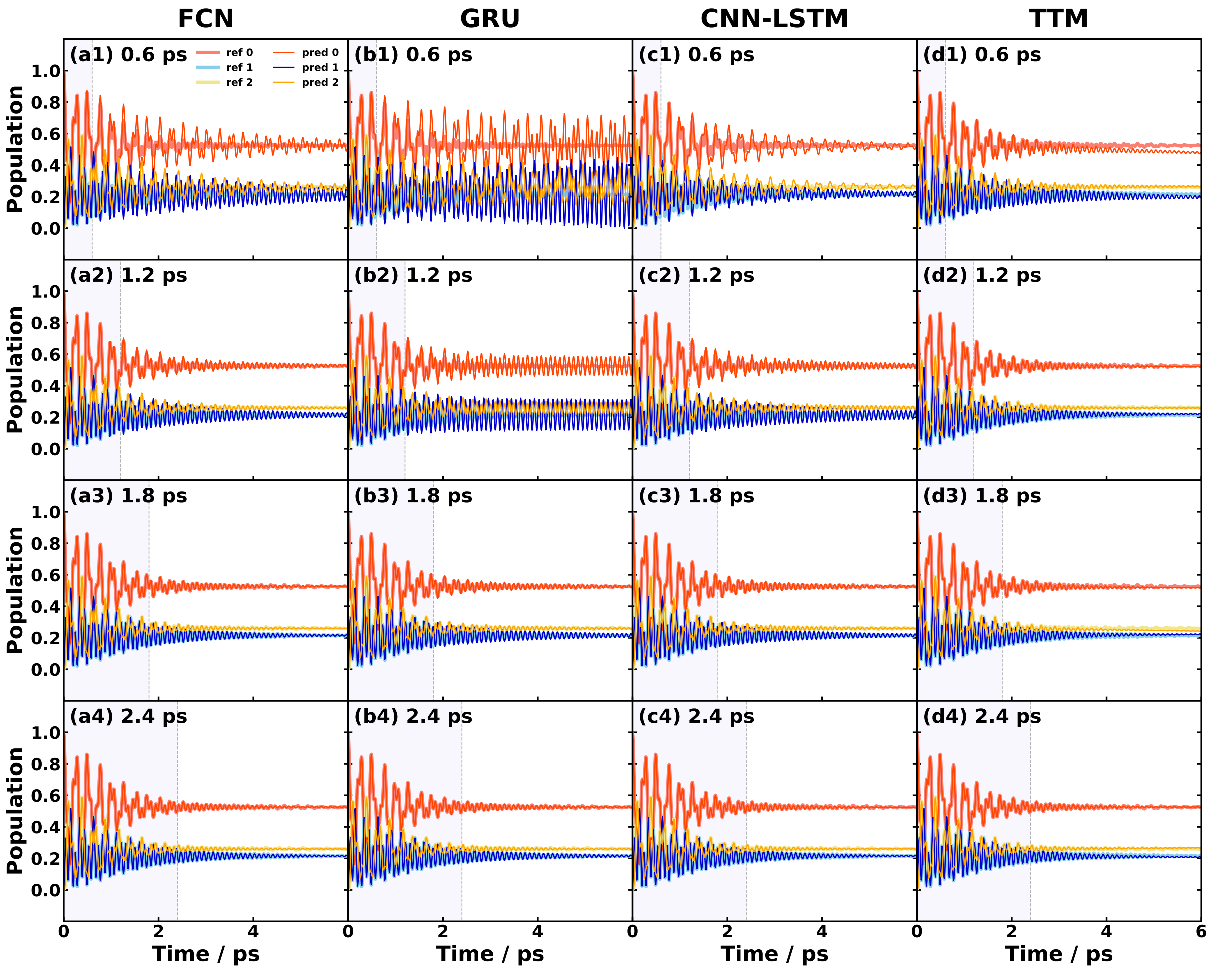

FIG. S10. SFL predictions for MSH #1 using different methods: (a) FCN, (b) GRU, (c) CNN-LSTM, and (d) TTM. The predicted long-time dynamics of the populations (thin lines) are compared with the reference RI-LSC2 dynamics (thick lines). Here, hyper-learning time is the last 20% of the learning time, which is indicated as the shaded area.

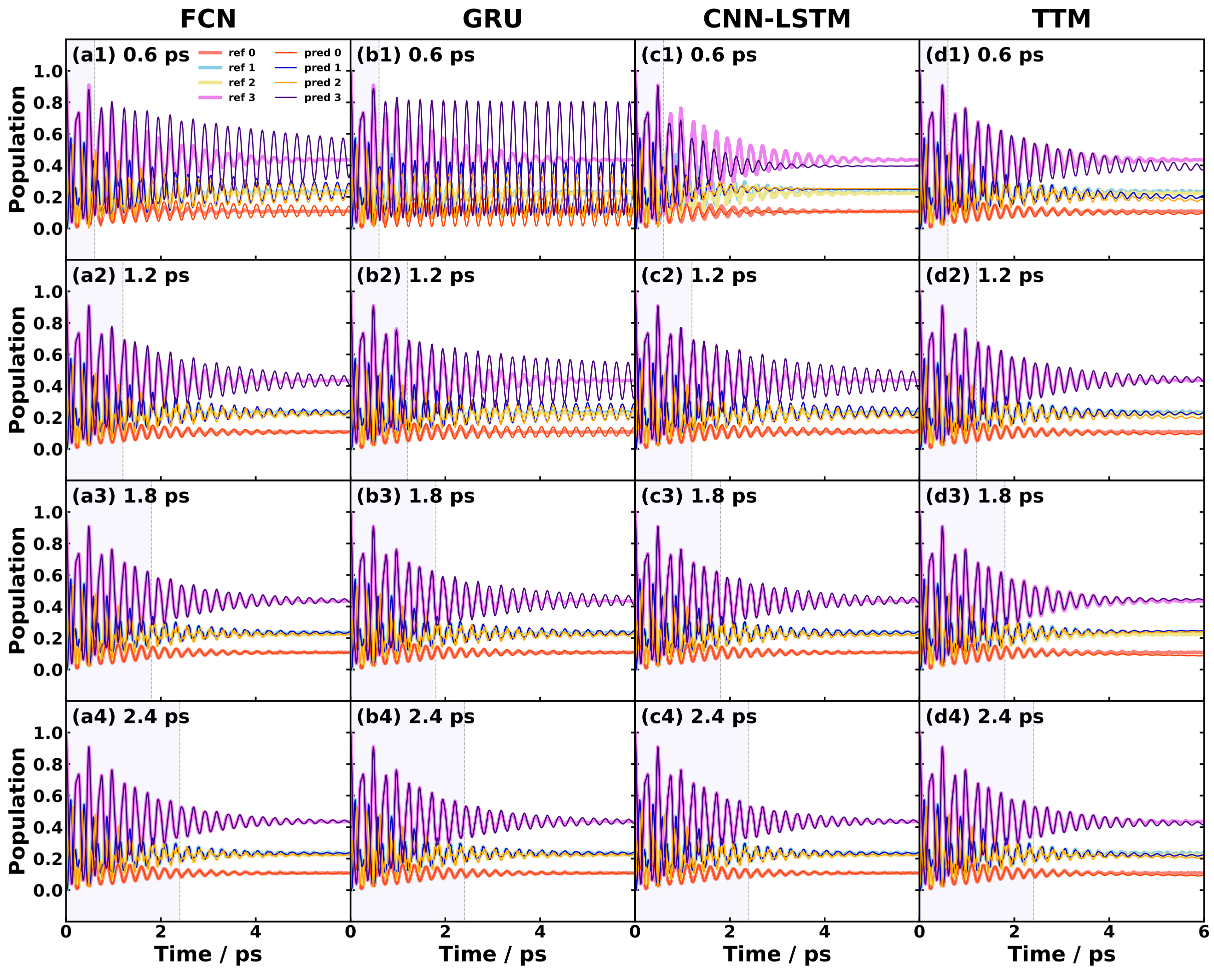

FIG. S11. SFL predictions for MSH #2 using different methods: (a) FCN, (b) GRU, (c) CNN-LSTM, and (d) TTM. The predicted long-time dynamics of the populations (thin lines) are compared with the reference RI-LSC2 dynamics (thick lines). Here, hyper-learning time is the last 20% of the learning time, which is indicated as the shaded area.

REFERENCES

[1] J. Cerrillo and J. Cao, “Non-Markovian Dynamical Maps: Numerical Processing of Open Quantum Trajectories,” Phys. Rev. Lett. **112**, 110401 (2014).

[2] Z. Hu, D. Brian, and X. Sun, “Multi-State Harmonic Models with Globally Shared Bath for Nonadiabatic Dynamics in the Condensed Phase,” J. Chem. Phys. **155**, 124105 (2021).

[3] Z. Hu and X. Sun, “All-Atom Nonadiabatic Semiclassical Mapping Dynamics for Photoinduced Charge Transfer of Organic Photovoltaic Molecules in Explicit Solvents,” J. Chem. Theory Comput. **18**, 5819–5836 (2022).

[4] Z. Liu, N. Lyu, Z. Hu, H. Zeng, V. S. Batista, and X. Sun, “Benchmarking Various Nonadiabatic Semiclassical Mapping Dynamics Methods with Tensor-Train Thermo-Field Dynamics,” J. Chem. Phys. **161**, 024102 (2024).

[5] X. Sun and E. Geva, “Non-Condon Equilibrium Fermi’s Golden Rule Electronic Transition Rate Constants via the Linearized Semiclassical Method,” J. Chem. Phys. **144**, 244105 (2016).
